# Beyond α‑GalCer: Medicinal Chemistry Insights Driving Structural Evolution of CD1d Ligands

**DOI:** 10.1021/acs.jmedchem.6c00783

**Published:** 2026-06-13

**Authors:** Emiliano Paradiso, Žiga Jakopin

**Affiliations:** Faculty of Pharmacy, 37663 University of Ljubljana, Ljubljana SI-1000, Slovenia

## Abstract

CD1d-restricted glycolipids have emerged as a cornerstone in the development of next-generation immunotherapies. This perspective provides a comprehensive update on structure–activity relationships, specifically examining how modifications of the prototypical CD1d ligand α-galactosylceramide (α-GalCer) to the galactosyl headgroup, phytosphingosine base, and fatty acyl chain dictate the modulation of invariant natural killer T (iNKT) cell responses. We explore the key pharmacophores and receptor interactions that polarize subsequent immune response toward either a pro-inflammatory Th1 or an anti-inflammatory Th2 phenotype. Building upon the structural evolution of sophisticated chemotypes, we evaluate the potential of these agonists in synergistic combinations with other adjuvants. Furthermore, we highlight the emerging transition toward fully synthetic self-adjuvanting vaccines, which ensure cellular colocalization and coordinated activation by covalently integrating antigens with glycolipid agonists. Collectively, these advancements underscore the transformative potential of tailored glycolipid design in engineering specific and durable immunity against cancer and infectious diseases.

## Graphical abstract

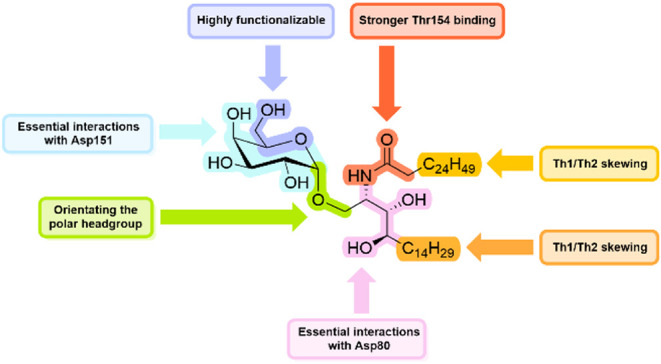

## 1 Introduction

The CD1 family comprises five antigen-presenting glycoproteins structurally and functionally related to major histocompatibility complex class I (MHC-I). These isoforms differ in their intracellular trafficking routes, enabling them to survey distinct endosomal compartments and present diverse lipid antigens to T cells.^1–4^ Based on sequence homology, the CD1 family is divided into two groups: group I (CD1a, CD1b, CD1c, CD1e) and group II (CD1d). While some mice strains can express two different codifying genes for CD1d,^5^ humans express just a single one, broadly distributed across antigen-presenting cells (APCs), intestinal epithelium, hepatocytes, B cells, and certain tumor cells, underscoring its role in mucosal immunity. Functionally, CD1d acts as an MHC-I-like glycoprotein specialized in presenting endogenous and exogenous glycolipids (i.e., glycolipid antigens) to invariant natural killer T cells (iNKT, also referred to as type I NKT), a unique, predominant subset of NKT cells characterized by a semi-invariant T-cell receptor (TCR), which comprise 0.01–1% of total circulating T cells in human and 0.5–2% in mice.^6–10^ CD1d surveillance includes the endoplasmic reticulum (ER), and the secretory and endocytic cell pathways, where it passively acquires the most abundant endogenous lipids available. In the ER, CD1d is predominantly loaded with phosphatidylcholine, while trafficking through endosomal compartments promotes exchange with sphingomyelin and lysophospholipids.^11^ These lipids occupy the CD1d binding groove to stabilize the molecule as it recycles through the cell, but do not activate the immune system pathway. In contrast, a potent endogenous agonism is attributed to α-linked glycosylceramides, notably α-galactosylceramide (α-GalCer, in the periphery) and α-glucosylceramide (α-GlcCer, for thymic selection), which are constitutively generated in mammalian cells and selectively presented to iNKT cells.^12^

Upon activation, iNKT cells rapidly release a broad spectrum of cytokines, most prominently interferonγ (IFNγ) and interleukin-4 (IL4), triggering a cascade that engages dendritic cells (DCs), natural killer (NK) cells, B cells, and both CD4^+^ and CD8^+^ T cells. This simultaneous induction of Th1- and Th2-type responses positions iNKT cells as pivotal orchestrators of immune regulation.^13^ Although murine iNKT cells are predominantly CD4^+^ or double-negative, human iNKT cells comprise a functionally distinct CD8^+^ subset. This subset variation is critical because wild-type mice are devoid of CD8^+^ iNKT cells.^14,15^ As human CD8^+^ iNKT cells are biased toward distinct pro-inflammatory Th1 responses, standard murine models fail to capture this essential immunological nuance.^16^ Directing this cytokine balance remains a central design objective, as selective polarization toward Th1 or Th2 immunity can profoundly influence therapeutic outcomes.^17–20^ Indeed, CD1d/iNKT interactions can be strategically tailored to favor host defense or tumor surveillance, or alternatively to mitigate autoimmunity and inflammatory/metabolic disorders.^21–23^ In this context, medicinal chemistry campaigns have focused on fine-tuning the CD1d binding and cytokine profiles through rational structural modifications. Over the past two decades, α-GalCer and its analogs have been extensively explored as immunomodulators. Reported strategies include amide isosteres, heteroaromatic moieties, fluorocarbonyl groups, covalent ligands, and even photoswitchable analogs, all aimed at optimizing ligand stability, receptor engagement, and functional selectivity. Importantly, by combining rational design with conjugation chemistry, recent advances in iNKT cell modulation have highlighted CD1d ligands as a powerful platform as vaccine adjuvants in cancer immunotherapy^22,24–26^ infectious^27^ and autoimmune diseases,^28^ and diagnostic tools for the precise study of the biological mechanism of action of such compounds.

## 2 CD1d Structure and Its Interplay within Innate and Adaptive Immunity

The CD1d/iNKT mechanism represents a unique concept in immunology where lipid antigens, rather than peptides, are presented to TCRs for immune recognition. The first crystal structure of mouse CD1d without a bound ligand revealed a deeper and narrower antigen-binding groove than MHC class I molecules, with two large hydrophobic pockets hypothesized to interact with alkyl chains of amphipathic lipids.^29^ This hypothesis was confirmed when the first human CD1d structure was solved in complex with α-GalCer in 2005 (PDB: IZT4),^1^ followed by structures of human CD1b and CD1a with defined ligands that provided detailed antigen-binding modes.^3^ As with classical MHC class I molecules, CD1d is formed by a single-pass noncovalently linked transmembrane heavy chain associated with soluble β2-microglobulin (β2m). While α1 is a continuous helix with 8 turns (29 amino acids 60–88), α2 is composed of two helical segments connected by a small 2- or 3-residue hinge and spans about 44 amino acids (139–182). The portal entrance is defined mainly by Asp79 and Asp80 in the α1 helix (Figure 1) and the hinge in the α2 helix. This structure shapes an antigen-binding cavity formed by the inner sides of the α1 and α2 helices and a floor settled by the β2m platform.

**Figure 1:**
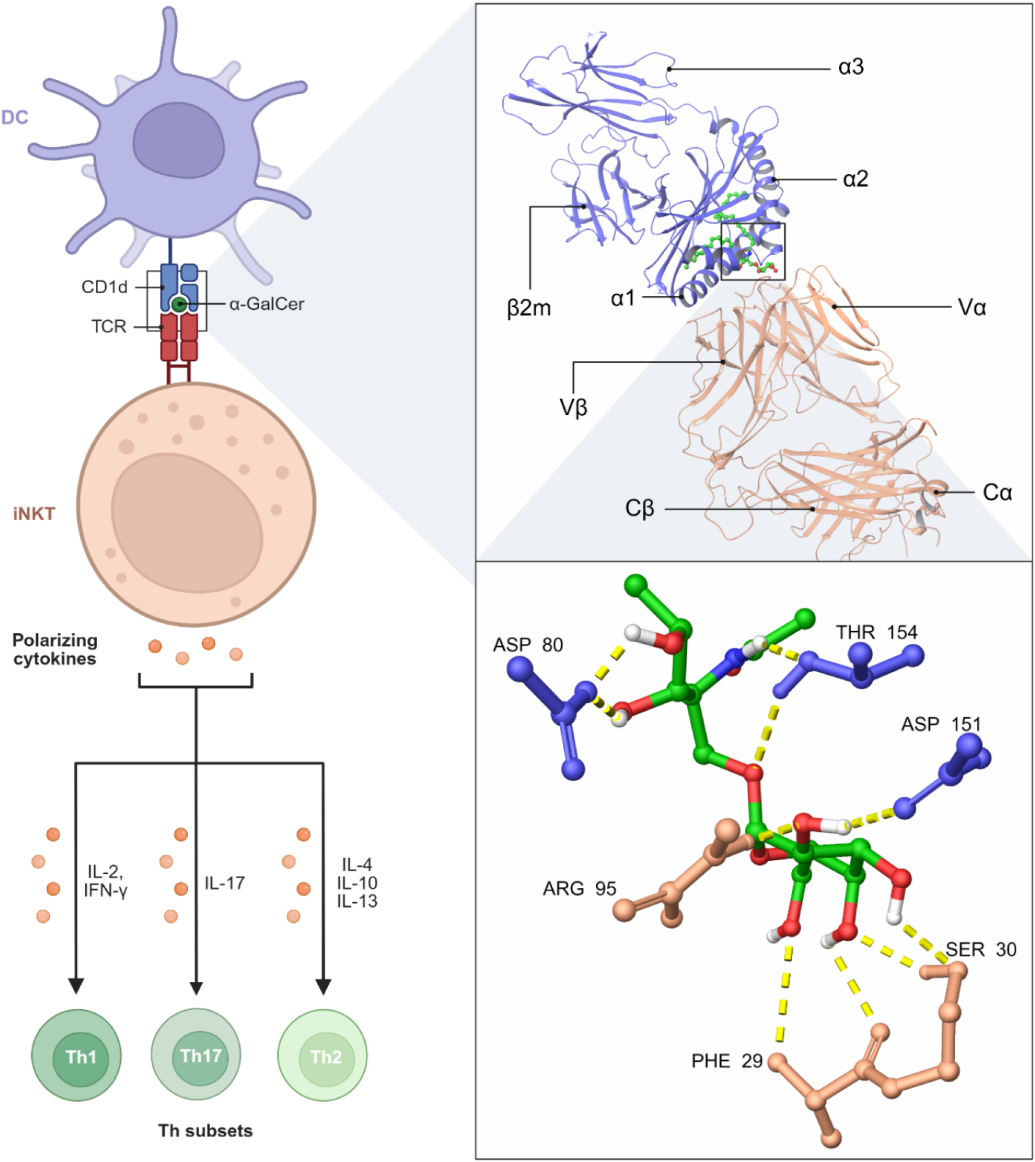
Cellular and molecular mechanisms^13^ of interactions of the immunological synapsis of CD1d/α-GalCer/iNKT (PDB: IZT4).^1^ Figure was created using Maestro (Schrödinger) and BioRender (https://BioRender.com/nj4dzk9).

As hypothesized, the antigen-binding groove consists of two hydrophobic channels designated A′ and F′ that accommodate lipid alkyl chains, with the A′ channel binding longer acyl chains up to 26 carbons and the F′ channel accommodating shorter chains up to 18 carbons. Both human and mouse CD1d were found to be well conserved overall (about 60% identity for hCD1d vs mCD1d1/2),^30^ but single amino acid changes in the α1/α2 helices reshape the groove and alter how specific lipids are oriented and recognized with a smaller lipid binding pocket that favors shorter lipids.^31,32^ A well-documented case is a glycine to tryptophan difference in the α2 helix (Gly in mouse, Trp153 in human) that prevents proper flattening and TCR recognition of iGb3 when bound to hCD1d, while the same lipid is antigenic in mCD1d, highlighting species selective lipid display.^33^ Mouse CD1d also interacts more strongly than human CD1d with the AP-3 adaptor, routing mCD1d more efficiently into late endosomes/lysosomes, which influences access to complex, processed glycolipids during iNKT selection and activation.^9^ For example, this Trp153 residue prevents the proper flattening and TCR recognition of iGb3 when bound to human CD1d, whereas the same lipid is highly antigenic in mice. Consequently, certain ligands may potently stimulate human iNKT cells *in vitro* but completely fail to activate murine iNKT cells *in vivo*.

The mechanism of lipid interaction orients amphipathic lipids, so their polar head groups protrude from the surface between the two α-helices, making the polar head the main portion exposed for TCR recognition. Crystal structures reveal that CD1d undergoes significant conformational changes upon lipid binding, with empty CD1d adopting a more open conformation and lipid-loaded CD1d assuming a closed conformation. CD1d is unique among CD1 isotypes in having three additional solvent-exposed tryptophans (Trp140, Trp153, Trp160) in the α2 helix that do not directly contact lipid antigens but serve as sensitive probes of ligand binding and conformational changes, and a unique Trp63 in the α1 helix. Molecular dynamics simulations reveal that these tryptophan residues display dynamic behavior sensitive to the type of ligand harbored in the hydrophobic channels and the type of polar headgroup exposed at the portal entrance.^34^ Trp140 exhibits considerable mobility in apo forms and when the F′ channel is empty or partially occupied but shows very low mobility when a lipid chain fills the F′ channel, suggesting that F′ channel occupancy induces external surface changes at the Trp140 region. Trp153, located at the portal entrance, shows large mobility values in complexes with single acyl chain ligands but low mobility in the α-GalCer complex due to the fixed position imposed by the bulky galactose moiety and complete channel filling. Within the groove itself, Trp40 is involved in lipid binding and acts as a lid that restricts acyl chain accommodation, while Trp131 contributes to shaping the inner wall of the cavity. These structural determinants define how synthetic ligands must balance chain occupancy with optimal headgroup exposure for TCR recognition and downstream immune activation.^34^

iNKT cells express a semi-invariant TCR composed of an invariant Vα14-Jα18 chain (Vα24-Jα18 in humans) paired with limited Vβ chains, predominantly Vβ8.2, Vβ7, and Vβ2 in mice. The Vβ bias results from differential affinities for CD1d/antigen complexes rather than pairing incompatibility, as all Vβ chains can pair with the invariant α chain, but only specific Vβ segments produce TCRs that interact effectively with CD1d/glycolipid complexes. The CDR2β loop dictates basal CD1d interaction while CDR3β sequences modulate overall avidity, with Vβ8.2 conferring higher avidity than Vβ7 and Vβ2. Structural studies reveal that iNKT TCRs dock on CD1d/lipid complexes in a unique parallel orientation distinct from conventional TCR-MHC interactions, with the invariant TCRα chain dominating through its CDR1α and CDR3α loops while the TCRβ chain contributes primarily through its CDR2β loop.^35^ The Vβ bias in iNKT cell repertoires results from differential affinities for CD1d/antigen complexes, with specific CDR2β residues mediating critical contacts with CD1d. The glycolipid ligand is engaged only by the invariant TCRα chain, while the TCRβ chain affects affinity by modulating CD1d binding ability.^36^

iNKT cells also express CD40L and can therefore induce activation of DCs, suggesting they function as helper T cells.^37,38^ The helper activity involves CD40 signaling, with activation of DCs also involving tumor necrosis factor (TNF) and type I and II IFNs. CD1d engagement by iNKT cells initiates Lck-dependent phosphorylation of CD3 ITAMs, promoting recruitment of the tyrosine kinase ZAP-70 and subsequent phosphorylation of the adaptor protein LAT. This event triggers the canonical intracellular signaling cascade, including activation of phospholipase C gamma (PLCγ), Ca^2+^ flux, calcineurin-dependent NFAT nuclear translocation, and parallel protein kinase C (PKC)-mediated activation of NF-κB and the Ras/MAPK/AP-1 pathway. In parallel, iNKT-derived IFN-γ and TNF engage DCs and macrophages to produce IL-12, which in turn feeds back to further activate iNKT and NK cells.^39^

Activation of iNKT cells *in vivo* with α-GalCer results in the simultaneous release of both Th1 and Th2 cytokines, resulting in the ability of iNKT cells either to enhance or to suppress immune responses. Activated iNKT cells can secrete a broad spectrum of cytokines, including ILs, chemokines, colony-stimulating factors, IFNs, and TNFα, influencing both innate and adaptive immune responses. The repertoire of cytokines produced is modulated by the strength of iNKT cell TCR signaling events, as well as by the type of APC presenting the iNKT cell agonists (Figure 2). For instance, IFNs and IL-12 produced by DCs promote pro-inflammatory M1 macrophages, NK-cell cytotoxicity and Th1 polarization of CD8^+^ T cells, while IL-4 and IL-13 provide a Th2 environment that modulates macrophage polarization toward the M2 phenotype to dampen excessive inflammation.^40,41^ Most B cells are also able to express α-GalCer-loaded CD1d on their surface, which allows for interaction with iNKT cells and promote B cell proliferation and antibody production.^42,43^ Furthermore, CD1d regulatory B cells are able to present CD1d/lipid complex to iNKT cells to induce an IFN-γ-producing, inflammation-limiting iNKT phenotype that helps to fine-tune the inflammatory response.^43^ At the same time, upon direct contact with iNKT cells, FOXP3 regulatory T cells (Tregs) suppress proliferation and cytokine production in the presence of bacterial CD1d-restricted glycolipids, upregulating FOXP3 and producing an IL10-rich environment able to enhance suppressive capacity of FOXP3 Tregs.^44^

**Figure 2:**
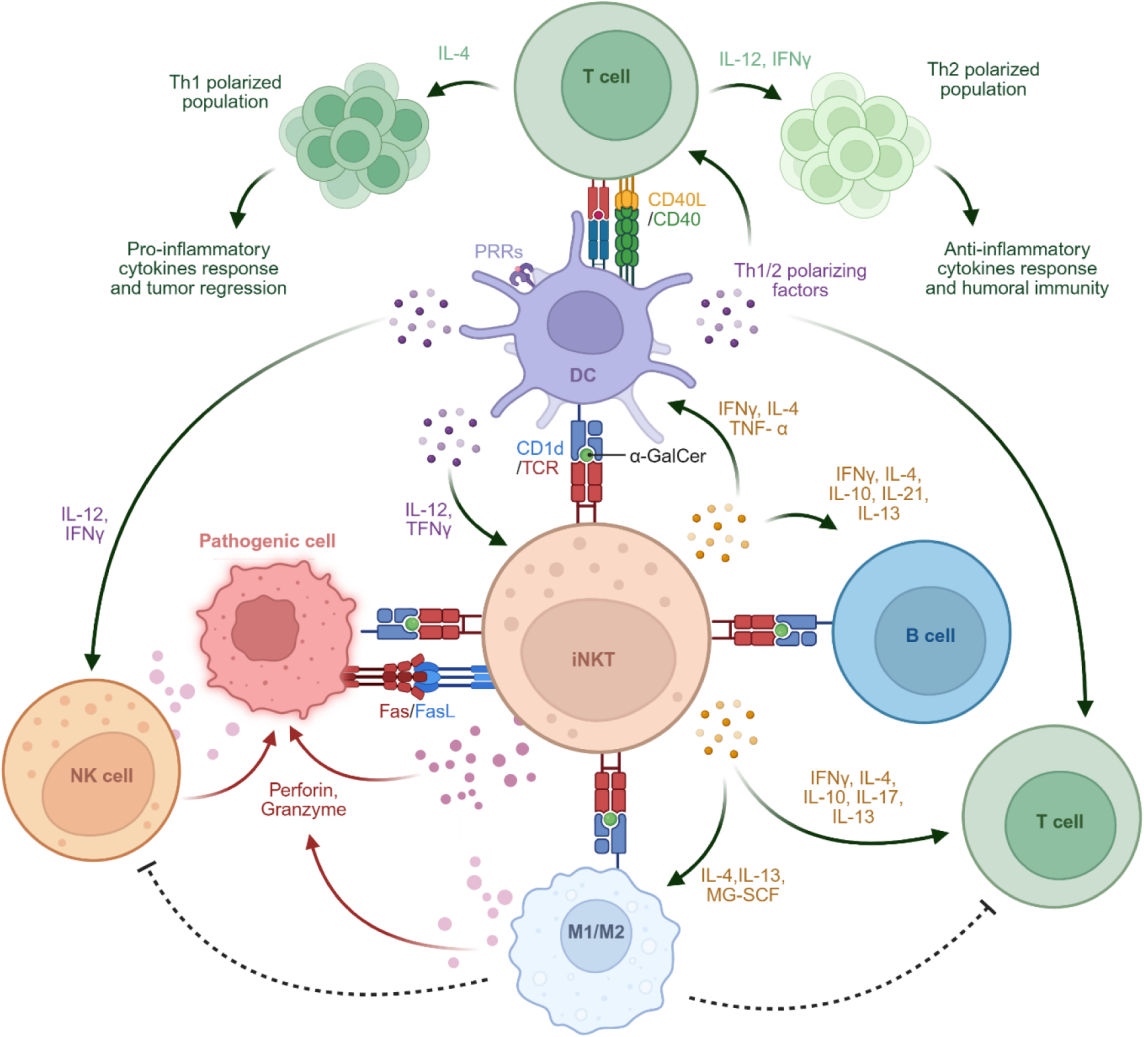
Schematic representation of the crosstalk among iNKT cell and the main immune system effector cells.^54^ Figure was created using BioRender (https://BioRender.com/42hz39m).

The kinetics of response to stimulation are notably different from conventional T cells, with iNKT cells exhibiting functional activity within hours of engagement, rather than several days for conventional T cells.^45^ This rapid response is facilitated by iNKT cells having stores of preformed cytokine mRNA enabling rapid release of large quantities upon TCR engagement. Rapid cytokine release by CD1d-restricted cells (such as IL-17-mediated neutrophil recruitment) can drive the immediate mobilization and activation of immune effector cells at sites of early infection, thus establishing a rapid and localized defensive response.

Interestingly, CD1d DCs integrate TCR-dependent CD40 signaling with pattern recognition receptors (PRRs), particularly Toll-like receptors (TLRs). CD40-TRAF signaling synergizes with MyD88-dependent TLR signaling to amplify NF-κB and IRF activation, promoting IL-12 and type I IFN production and underlining iNKT cells as rapid innate-like sensors that bridge PRR-driven immunity with adaptive responses.^39,46,47^ This leads to further increases in the expression of MHC molecules and costimulatory molecules, and significantly enhanced production of key cytokines such as IL-12, that helps drive T cell differentiation. It also provides an explanation as to how microbial cues or adjuvants reshape iNKT cell responses *in vivo*. Murine iNKT cells can upregulate TLR3, TLR5, TLR7, TLR9 after strong TCR stimulation with α-GalCer and then respond to their ligands with enhanced IFN-γ, IL-4, TNFα, amplifying downstream macrophage activation and antiviral functions.^48,49^

Since iNKT cells can act as universal helper T cells capable of licensing B cells (triggering maturation, class switching, higher antibody titers, and memory pool expansion) independently of conventional CD4^+^ T cell help,^28,50,51^ exploitation of this pathway in vaccine design has led to the development of codelivery strategies of different antigens with α-GalCer, ensuring copresentation of both the glycolipid ligand and the target antigen on the same APC or B cell surface.^52,53^ Unlike conventional TLR agonists, which amplify adaptive immunity indirectly through DC maturation and cytokine-mediated Th1/Th2 polarization, skewing antibody isotypes toward the cytotoxicity-associated IgG2*a*/2b subclasses elicit a hybrid humoral phenotype encompassing extrafollicular plasmablast differentiation, germinal center formation, affinity maturation, and robust primary IgG1 responses, even in MHC class II-deficient hosts.^52^

Besides their role as immunomodulatory conduits, iNKT cells can also enhance the immune function against tumors by direct cytotoxicity via the Fas/FasL pathway,^55^ and the release of perforin and granzyme B which induce apoptosis in CD1d-expressing tumor cells (Figure 2). Upon activation via their invariant TCR, iNKT cells rapidly express CD40L, which interacts with CD40 on DCs. This interaction triggers DC maturation and the subsequent secretion of IL-12. The latter, in a reciprocal feedback loop, stimulates iNKT cells to produce massive quantities of IFN-γ. This burst of IFN-γ is a critical catalyst for trans-activation: it promotes the recruitment and activation of NK cells and cytotoxic T lymphocytes (CTLs), enhancing their tumor-killing capacity. Finally, iNKT cells can reprogram the tumor microenvironment (TME) by inhibiting the recruitment of myeloid-derived suppressor cells (MDSCs) and shifting macrophage polarization from a pro-tumor M2 phenotype to an antitumor M1 phenotype.

## 3 Structural Evolution and Structure–Activity Relationships of CD1d Ligands

Despite their relatively limited antigen receptor repertoire, CD1d-restricted iNKT cells recognize a remarkably diverse range of lipidic antigens. The earliest CD1d ligands, comprising 177 lipid species across glycerophospholipids and sphingolipids, all shared a typical amphipathic architecture in which a polar headgroup is covalently linked to one or more aliphatic lipid chains.^56^ α-GalCer (also known as KRN7000) was identified as the immunostimulatory component of the marine sponge *Agelas mauritianus*, and remains the most potent agonist of the iNKT cell TCR. The development of a high-yield synthetic route provided the first well characterized ligand that could be used to identify and activate virtually the entire population of iNKT cells.^54,57,58^ It has shown broad preclinical efficacy against multiple cancers (melanoma, liver, colon) and in autoimmune and infectious models,^59^ and is increasingly used as a potent adjuvant in vaccines, including intranasal influenza vaccines, HIV peptide vaccines, and lipid-nanoparticle based mRNA cancer as well as antibacterial vaccines, where it enhances both antibody and CD8 T-cell responses and long-term protection.^60–63^

Structural analysis (shown in Figure 3) reveals that the glycolipid fits tightly within the CD1d binding groove with its 26-carbon acyl chain occupying the A′ channel and 18-carbon phytosphingosine chain (also known as (4*R*)-hydroxysphinganine) filling the F′ channel.^1,34,64^ The galactose headgroup is positioned for optimal TCR recognition through hydrogen bonding interactions between the surface residues of CD1d and the hydroxyl groups of the galactose and phytosphingosine portion, which can be considered crucial for maintaining α-GalCer in the correct position and orientation for recognition by the TCR. Unlike most mammalian ceramide glycolipids which contain proximal sugars in the β-anomeric form, in α-GalCer the sugar moiety is linked in the α-anomeric configuration. This allows several hydrogen-bonding interactions between the surface residues of CD1d and the hydroxyl groups of the galactose and phytosphingosine base, with the 2″–OH and 3″–OH hydroxyl groups of the D-galactose ring hydrogen bonded to the two Asp151 oxygen atoms, and the 3′–OH and 4′–OH groups of the phytosphingosine chain interacting with the Asp80 residue.^65^ These bonds anchor α-GalCer in a distinct orientation and place it correctly in the lipid-binding groove, which directly affect the loading and tighter fit in A′ and F′ pockets, and therefore strength of binding.^66,67^ Stereochemistry studies indicate that the tridimensional spatial orientations of the amide group and (3′*S*)–OH group of phytosphingosine are crucial, and the configuration of the amide group is much more important than that of 3′–OH group. On the other hand, the presence of the 4′–OH group is important for optimal TCR recognition, but its specific stereochemistry is not strictly constrained. Synthetic inversion of the C′4 stereocenter from (4′*R*) to (4′*S*) results in an analog that maintains comparable potency to α-GalCer in both cellular proliferation and the secretion of Th1 and Th2 cytokines.^68,69^

**Figure 3:**
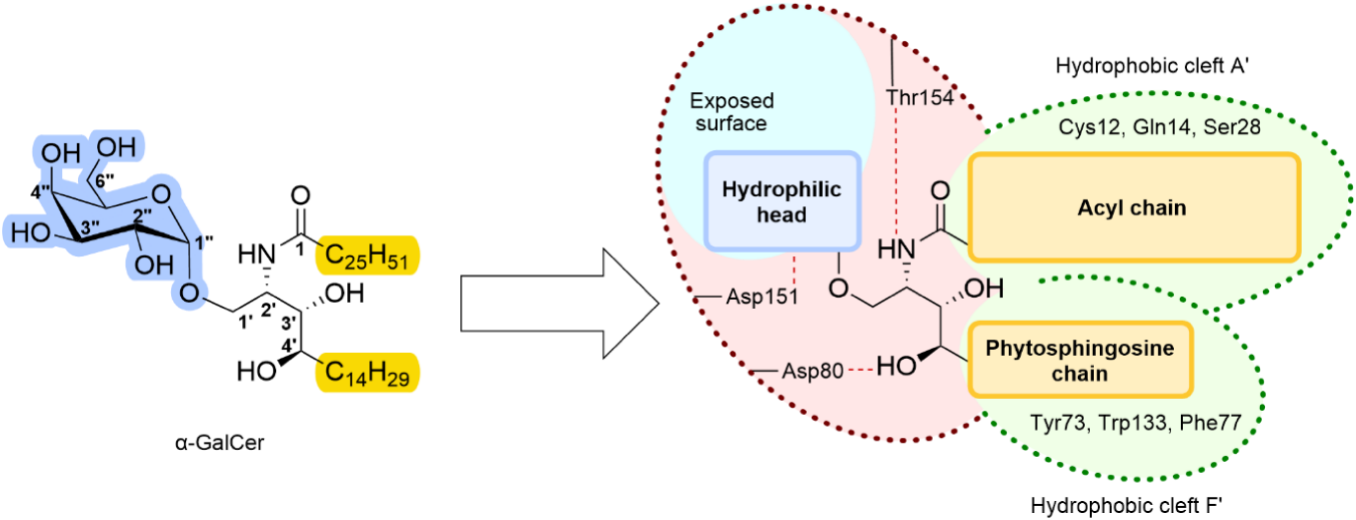
Schematic representation of α-GalCer and its binding site. The hydrophilic head protrudes outside the protein, allowing the exposure of galactose to interact with the essential amino acids of the iNKT TCR. Figure was created using ChemDraw.

In a clinical setting, αGalCer-pulsed APCs have been well tolerated in phase I–II trials in solid tumors and advanced nonsmallcell lung cancer (NSCLC), with no dose-limiting toxicity or severe treatment-related adverse events, and evidence of increased NK cells, IFNγ-producing cells and effector CD8^+^ T cells.^70–72^ However, several issues have emerged which prompted the search for substitutes or improved analogs: (i) due to the high amphipathic behavior, the molecule is essentially insoluble in water and barely dissolves in DMSO, often requiring specialized preparation techniques such as heating to 80 °C and prolonged sonication to obtain reproducible results. This lack of “pharmaceutical tractability” often necessitates the use of detergents or complex liposomal formulations, which can raise safety concerns or interfere with the immunological properties of vaccines; (ii) repeated intravenous or intraperitoneal injections of unformulated αGalCer can induce iNKT cell anergy, leading to hyporesponsive iNKT cells and potentially blunting adaptive immunity, requiring alternative routes (oral or intranasal) or *ex vivo* αGalCer-loaded dendritic cells to avoid this problem;^62^ (iii) its intrinsically mixed Th1/Th2 cytokine profile may be suboptimal in pathological settings where a selectively polarized Th1 response is required;^73,74^ and (iv) α-GalCer shows potent antitumor effects in mice^75–77^ but only minimal or suboptimal efficacy in human trials, explicitly attributed to interspecies differences in CD1d/iNKT systems.^9,15,26^ While generally safe, these differences in frequency, subsets, cytokine profiles, and tissue distribution are nontrivial and a major reason why mouse model *in vivo* results have been hard to translate into clinical efficacy (Rotolo et al., 2025; Shen et al., 2020; Zhang et al., 2019; Yang et al., 2015), with limited tumor regression and challenges in achieving effective tumor-targeted delivery and optimal iNKT engagement.^63,71,74^

Consequently, improved formulations, CD1d-targeted delivery systems, and next-generation glycolipids are being actively pursued to enhance immunomodulatory effects without adding significant toxicity. To facilitate the rational design of these novel compounds, a deep and up-to-date understanding of the molecular interactions is required. Previous Structure–Activity Relationship (SAR) analyses established a molecular framework for understanding how structural variations in the α-GalCer scaffold could selectively switch iNKT cell responses between the pro-inflammatory Th1 and anti-inflammatory Th2 profiles.^17–20^ While these studies identified a comprehensive molecular basis for cytokine polarization, the field has significantly progressed in exploring more sophisticated chemotypes to increase the binding affinity toward CD1d, stabilize the ternary complex, and create platform for the codelivery of multiple agonists. Consequently, in the next chapter we examine the new developments in CD1d agonism through a medicinal chemistry lens, and how those modifications are driving the structural evolution of CD1d ligands toward clinical practice.

### 3.1 Acyl Chain Modifications

#### 3.1.1 Acyl Chain Length

The length of the acyl chain is a primary determinant of how the ligand is anchored within the CD1d A′ pocket. Systematic variation of acyl chain length has established that the A′ pocket of CD1d accommodates a wide range of acyl chain homologues, although the acyl chain length governs both binding affinity and immunological outcomes of iNKT cell activation (Table 1).

**Table 1:** SAR of α-GalCer Acyl Chain Length Variations

**Acyl chain length**	**CD1d A′ pocket occupancy**	**Potency and cytokine bias**	**Mechanistic consequences**
Extra-short (C8–C10)	Very poor (less than 50% occupancy)	Very weak, but strongly Th2 biased	Highly unstable CD1d complex; failure of F′-roof formation
Short (C11–C14)	Poor	Weak, but strongly Th2 biased	Fast dissociation due to incomplete anchoring; can be overcome by polar or aromatic termini
Short-to-medium (C15–C18)	Partial	Low to moderate, modest Th2 biased	Rapid IL-4 release dominates
Medium-to-long (C20–C24)	Incomplete but effective	Moderate to high, with modest Th2 bias	Sufficient hydrophobic volume preserves TCR engagement
α-GalCer (C26)	Complete occupancy	High activity, mixed Th1/Th2 response	Complete A′-pocket filling yields highest complex stability
Extra-long (C27–C29)	Steric clash	Very weak, suppressed IFN-γ production	A′ pocket inability to accommodate longer chain

Truncation of the acyl chain, such as in compound **1** (PBS-25, C8:0; shown in Figure 4), results in a less stable CD1d/ligand complex, with nearly 100-fold reduction in global cytokine production compared to α-GalCer and specifically toward IFN-γ production.^78,79^ The residence time of these analogs has been related to the length of the hydrocarbon chains and the manner in which they fill the binding groove, with longer chains fitting well into the A′ providing for increased half-lives for APCs stimulation, with optimal activity typically observed for chains of 20–26 carbons.^64,80^ Compound **2** (C20:0; shown in Figure 4) represents a balanced structural modification with respect to acyl chain length and Th polarization, exhibiting enhanced Th2 bias relative to full-length α-GalCer while maintaining biological although reduced activity. This bias is thought to reflect a half-life threshold where the less stable CD1d/ligand complex provides enough stimulation for the rapid release of IL-4 but falls short of the sustained TCR engagement required for substantial IFN-γ production.^69^ Importantly, a broad range of acyl modifications have been explored that effectively compensated the loss of stability associated with acyl-chain truncation, including the introduction of conformational constraints, heteroatom substitution, and alternative aromatic or lipophilic motifs capable of restoring productive CD1d/TCR interactions without increasing overall chain length.

**Figure 4:**
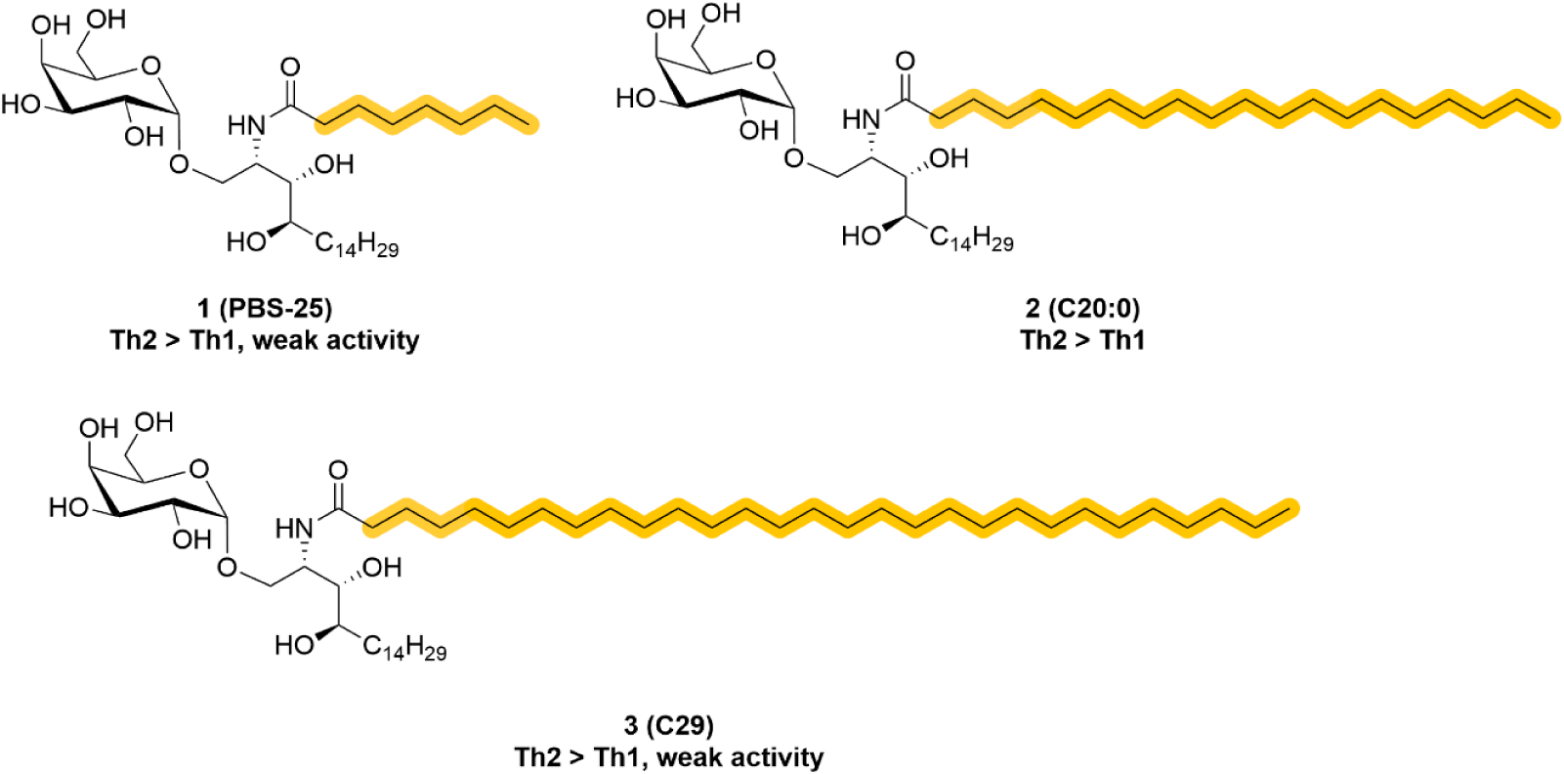
Structural modifications of the acyl chain in compounds **1**–**3** and their polarization capacity of immune response.

Chains longer than 26 carbons also produce a drastic loss of ability to induce IFN-γ production as evidenced by compound **3** (C29, Figure 4). This is because the A′ pocket is unable to accommodate alkyl chains longer than C26, resulting in steric clashes that destabilize the engagement with TCR.

#### 3.1.2 Acyl Chain Unsaturation

The introduction of double bonds into the fatty acyl chain of α-GalCer analogs significantly impacts the stability of CD1d/lipid complexes and promotes a bias toward Th2 responses.^81^ A notable example is provided by the α-GalCer analog **4** (Figure 5, C20:2), containing an 11,14-cis-diunsaturated C20 fatty acid which potently induces a Th2-biased cytokine profile, directly diminishing IFN-γ production and IFN-γ-producing iNKT-cell expansion.^64^ Mechanistically, the rate of dissociation for the saturated compound C20:0 is 2.15-fold faster than that of C20:2 (with half-lives of 170 and 367 min, respectively), indicating that the presence of unsaturation elements at carbons 11 and 14 favors the formation of more stable CD1d/lipid complexes. This stability is attributed to a preformed twist in the acyl chain caused by the double bonds, which facilitates the tightly curved conformation required to circumnavigate the central pole formed by Cys12 and Phe70 within the CD1d A′ channel. A slightly shorter unsaturated analog, C18:2 (Figure 5, compound **5**), has been shown to exhibit a stimulatory potency similar to that of C20:2, confirming that the unsaturation is a prominent structural feature for recovering the activity of truncated chains while maintaining a Th2 skew. This idea of unsaturation-induced Th2 bias is further exemplified by the polyunsaturated analog C20:4 (Figure 5, compound **6**), which induces a systemic cytokine pattern resembling OCH (the gold standard of Th2-polarizing CD1d ligands, shown in Figure 14), characterized by a rapid burst of serum IL-4 but a complete lack of the delayed and sustained IFN-γ production typically seen with α-GalCer.

**Figure 5:**
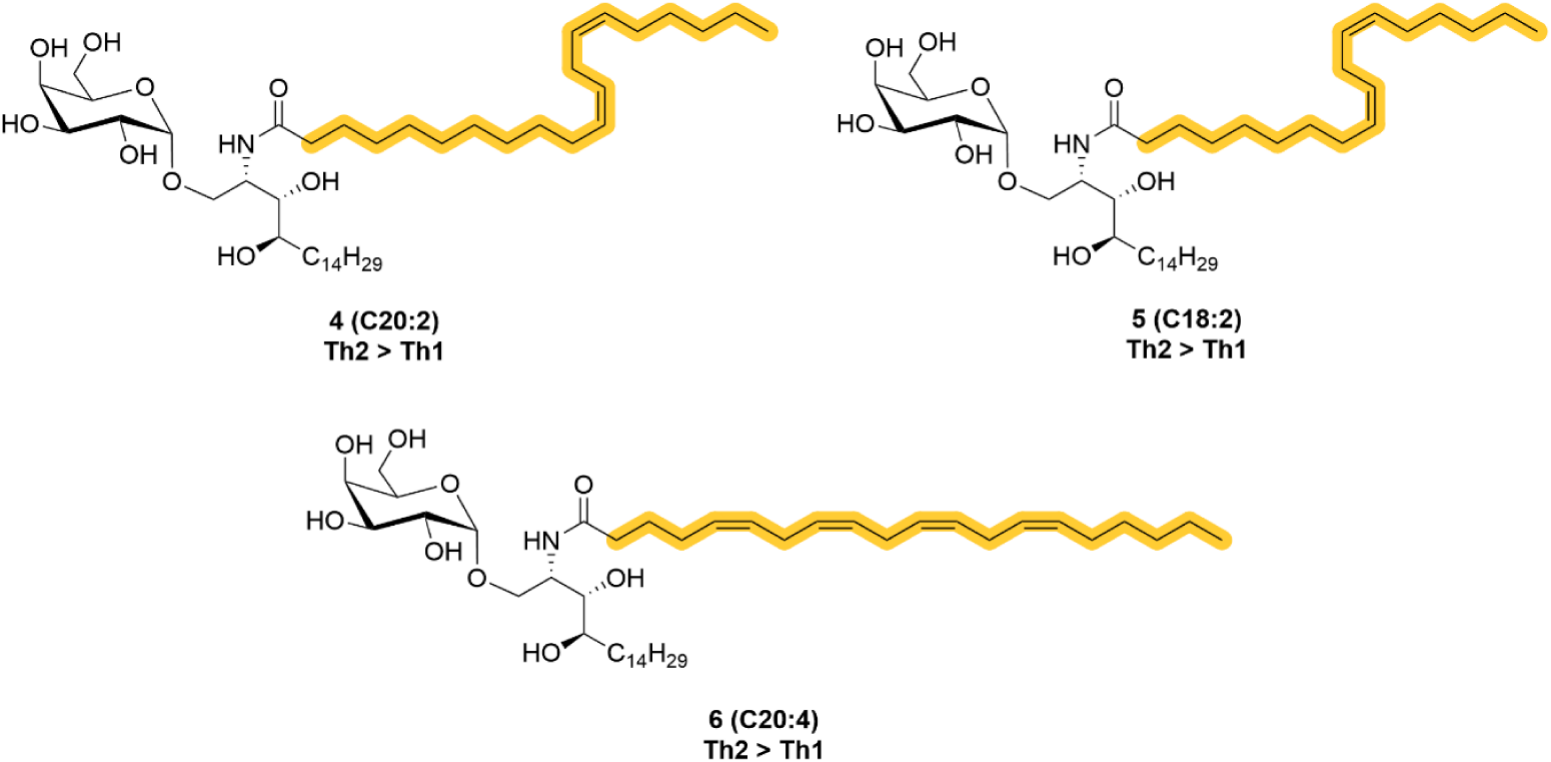
Structural modifications of the acyl chain in compounds **4–6** and their polarization capacity of immune response.

The alteration of the biological activity of such analogs indicates that the curvature of the acyl chain significantly affects the complementarity of the CD1d binding groove with the TCR. This is also reflected in the mechanism of cellular activation: administration of **4** resulted in a more rapid but less sustained CD69 up-regulation in NK and B cells, as well as a lack of substantial iNKT cell expansion.^82^ After *in vivo* administration of **4**, only a minimal transient expansion was observed on day 2, with no further expansion observable by day 5.

#### 3.1.3 Covalent Binders of the A′ Pocket

A possible strategy to circumvent the intrinsic limitation of truncated acyl chains involves the design of covalent-binding α-GalCer analogs, wherein the irreversible anchoring within the CD1d A′ pocket compensates for the attenuated interactions normally associated with shortened lipid tails. Chloroacetylamide analog (compound **7**, Figure 6) shows a 20-fold increase in cytokine production compared to the corresponding noncovalent analog, effectively compensating for the truncation of the acyl chain. This enhanced activity resulted from the formation of a more stable complex through covalent bond formation with Cys12 in the A′ pocket, with an overall improvement of the cytokine profile compared to that of α-GalCer.^83^ The formation of covalent bonds extends the residence time of ligands on CD1d, enabling enhanced iNKT cell activation even with suboptimal chain length. Despite the increased stability afforded by the covalent bond, these ligands consistently exhibited a Th2-biasing response. It has been proposed that the lack of filling the total volume of the CD1d binding groove causes a partial collapse or “settling” of the CD1d α helices. A classic example is the Phe84 residue on the α1 helix; in truncated models like OCH, this residue shifts significantly down into the empty groove. This distortion is allosterically transmitted to the surface, altering the positioning of critical residues (Glu83, Lys86, Arg89) that form the TCR recognition footprint.^69^ Nevertheless, this approach could have particular relevance for vaccine applications where sustained antigen presentation may be advantageous.

**Figure 6:**
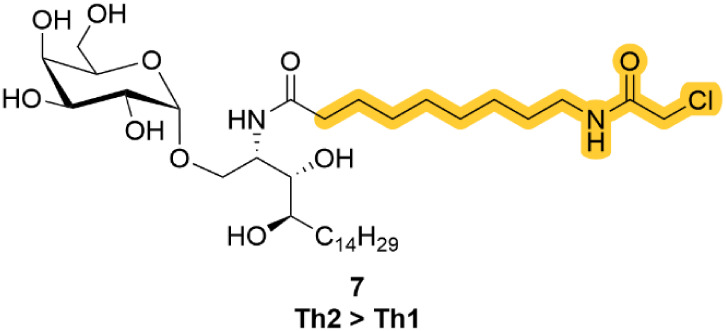
Structural modifications of the acyl chain in compound **7** and its polarization capacity of immune response.

#### 3.1.4 Branched Acyl Chain Derivatives

Compound KBC-009 (compound **8**, Figure 7) represents a viable solution to the challenge of poor solubility α-GalCer, which is one of the main hindrances for its pharmaceutical applications. This analog incorporates branched *N*-alkyl chains that enhance aqueous solubility while maintaining immunological activity.^84^ In nasal influenza vaccine studies, KBC-009 demonstrates substantial Th2-biasing adjuvant effects compared to inactivated virus alone, and to effectively boost the generation of CTLs, validating its therapeutic utility. The improved solubility addresses a key limitation of many CD1d ligands, which frequently necessitate complex formulations due to their high hydrophobicity, making them essentially insoluble in water. Increased aqueous solubility not only facilitates pharmaceutical development but also expands the feasibility of alternative administration routes, particularly for mucosal vaccination.

**Figure 7:**
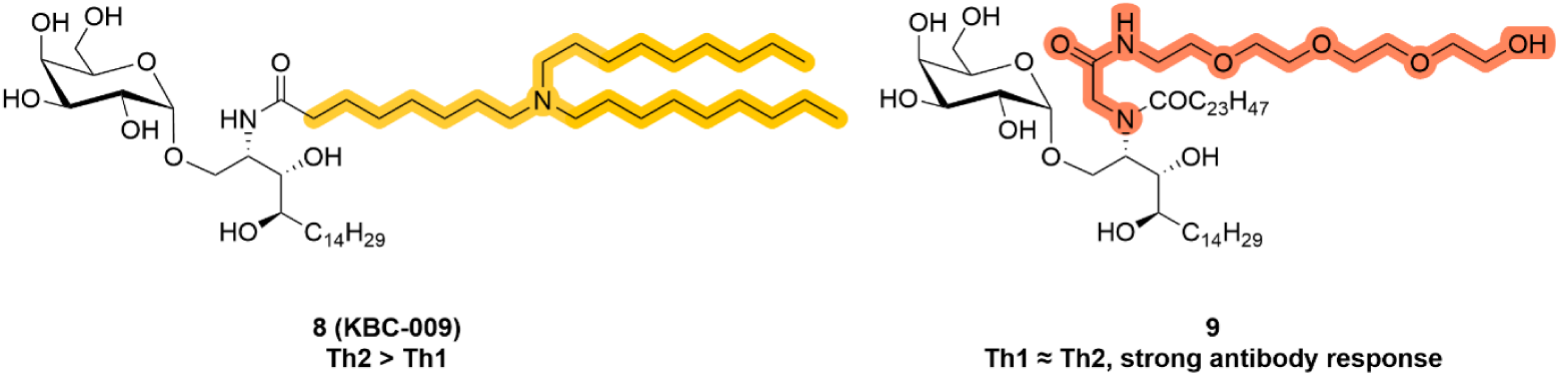
Structural modifications of the acyl chain in compounds **8** and **9** as well as their polarization capacity of immune response.

Recent advancements have identified a novel diversification hotspot at the amide bond nitrogen to improve solubility (compound **9**, Figure 7). Researchers have generated large libraries of multicomponent α-GalCer analogs with PEGylated functionalities to improve antigen-specific T-cell and antibody responses in parenteral and mucosal vaccines, outperforming the prototypical α-GalCer.^85^ Interestingly, the PEGylation tags can also be exploited as handles for future conjugation. Branched-chain modifications thus constitute a generalizable design principle for enhancing the pharmaceutical tractability of CD1d-restricted glycolipids while preserving essential bioactivity, with broad implications for their clinical advancement.

In addition to simple branching, it has been reported that the conformational restriction guided by the spatial architecture of the CD1d binding groove can clasp a central amino acid pole formed by residues Phe70 and Cys12 within the CD1d A′ pocket with high specificity.^86^ By occupying the binding pocket from two directions simultaneously, these branched analogs achieve a more optimal fit and significantly limit the torsional flexibility of the lipid tail. This conformational restriction reduces the entropic penalty typically associated with ligand binding, resulting in a more stable and sustained formation of the glycolipid/CD1d complex on the cell surface. Compound GCB-27a (compound **10**, Figure 8) has shown that this conformational restriction leads to highly potent Th1-biased responses, with superior antitumor efficacy and activity in both murine and human iNKT cells. As for the prototypical α-GalCer, the polarization is correlated with the length of the branched chains, as shorter analogs display very low Th1 bias (compound **11**, Figure 8).

**Figure 8:**
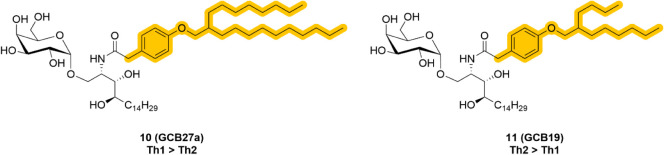
Structural modifications of the acyl chain in compounds **10** and **11** as well as their polarization capacity of immune response.

#### 3.1.5 Bioisosteric Replacements to the Amide Bond

Thioamide analogs represent the most extensively studied single-atom substitution, where the amide carbonyl oxygen is replaced with sulfur. Thioamides like DB06-1 (compound **12**, Figure 9) are superior hydrogen-bond donors than amides due to their increased polarity and N–H acidity.^87,88^ Cytokine polarization studies demonstrate that, while these modifications are not necessarily more effective *in vitro*, they generally exhibited increased IFN-γ production *in vivo* and promoted Th1-biased responses compared to their parent compounds,^89^ as was speculated that the sulfur allows for more intimate contact with CD1d Thr154 and increase the stability of the CD1d/glycolipid complex.^88^

**Figure 9:**
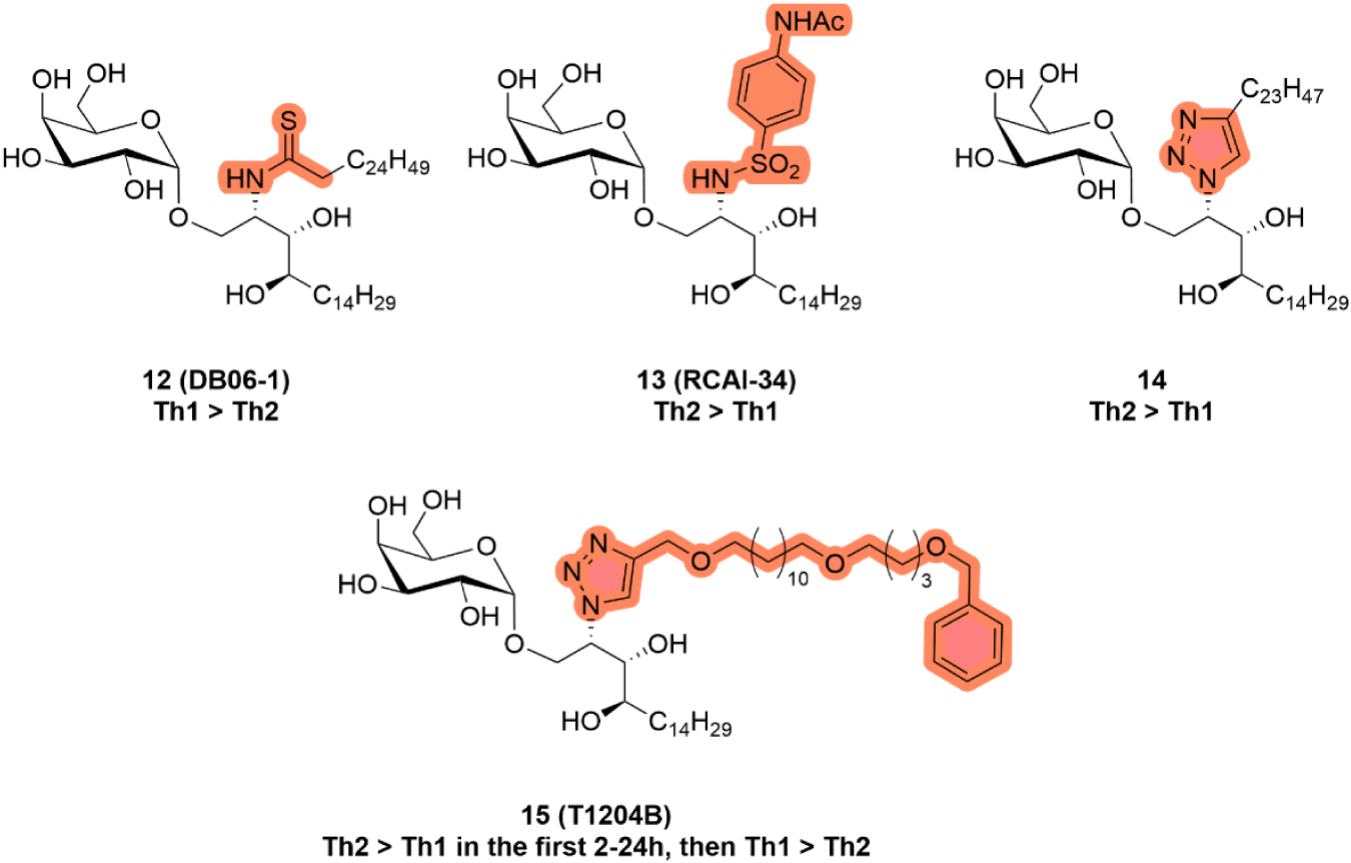
Structural modifications of the amide linkage in compounds **12**–**15** and their polarization capacity of immune response.

Carbamate analogs have also demonstrated significant potential by enhancing IFN-γ production compared to α-GalCer, while demonstrating unique iNKT transactivation properties through improved cytokine signaling or prolonged antigen presentation.^87^ Conversely, urea analogs and aryl- or alkylsulfonamide derivatives^90^ exposed the limitations of certain isosteric replacements, exhibiting weak Th2 stimulation only at high concentrations and poor overall activity, with the sole exception of compound **13**, (RCAI-34, Figure 9). The observed reduced activity likely reflects that additional hydrogen bonds at the amide position the precise disrupt binding into CD1d binding site and the presentation footprint required for CD1d/iNKT engagement. Similarly, ester and ether analogs show significant activity limitations, with ester derivatives exhibiting weaker cytokine responses and minimal IFN-γ production, while ether analogs completely lack stimulatory activity.^17,91^

Bioisosteric replacement of the amide group with a 1,2,3-triazole (compound **14**, Figure 9) has also been a viable option to apply synthetically accessible copper-catalyzed azide–alkyne cycloaddition (CuAAC) and click chemistry principles to immunomodulator design. This simple triazole analog increased the IL-4 versus IFN-γ bias by interacting with His158, shifting the immune response toward Th2 polarization.^92^ Systematic chain length optimization confirms that, while the triazole itself is able to skew the bias toward Th2 phenotype, the length of the chain itself is responsible for the potency of iNKT cell activation. Further refinement also led to a second-generation of triazole analogs, exemplified by T1204B, (compound **15**, Figure 9) which incorporates a terminal benzyloxy moiety tethered via a polyether spacer to target Trp181. This hybrid scaffold introduces a unique time-dependent switch in polarization: early stimulation (2–12 h) triggers a rapid Th2-skewed response characterized by high IL-4 levels; however, prolonged exposure leads to a progressive increase in IFN-γ secretion, resulting in a complete reversal of the cytokine bias toward a Th1-dominant profile by 48 h. This kinetic switch demonstrates that the inclusion of aromatic directing groups can effectively tune the immune polarization.^93^

#### 3.1.6 Heteroatom Modifications in the Chain

α-GalCer derivatives incorporating a diether moiety within the acyl chain have been rationally designed to target the few hydrophilic residues located deep within the otherwise hydrophobic CD1d A′ pocket. By systematically replacing methylene units with oxygen atoms at positions close to Cys12, Ser28, and Cys168, these analogs establish additional noncovalent hydrogen-bonding interactions that stabilize the CD1d/ligand binary complex without disrupting the essential hydrophobic contacts required for the binding in the A′ pocket. *In vitro* biological evaluations of compound **16** (Figure 10) have demonstrated to elicit IFN-γ and IL-4 patterns comparable to the parent compound, while being also able to increase the secretion of IL-17, suggesting a potential for enhancing host defense against extracellular pathogens.^94,95^ Conversely, compound **17** (Figure 10) of the same series drastically reduced the IL-17 release, indicating a specialized anti-inflammatory profile.

**Figure 10:**
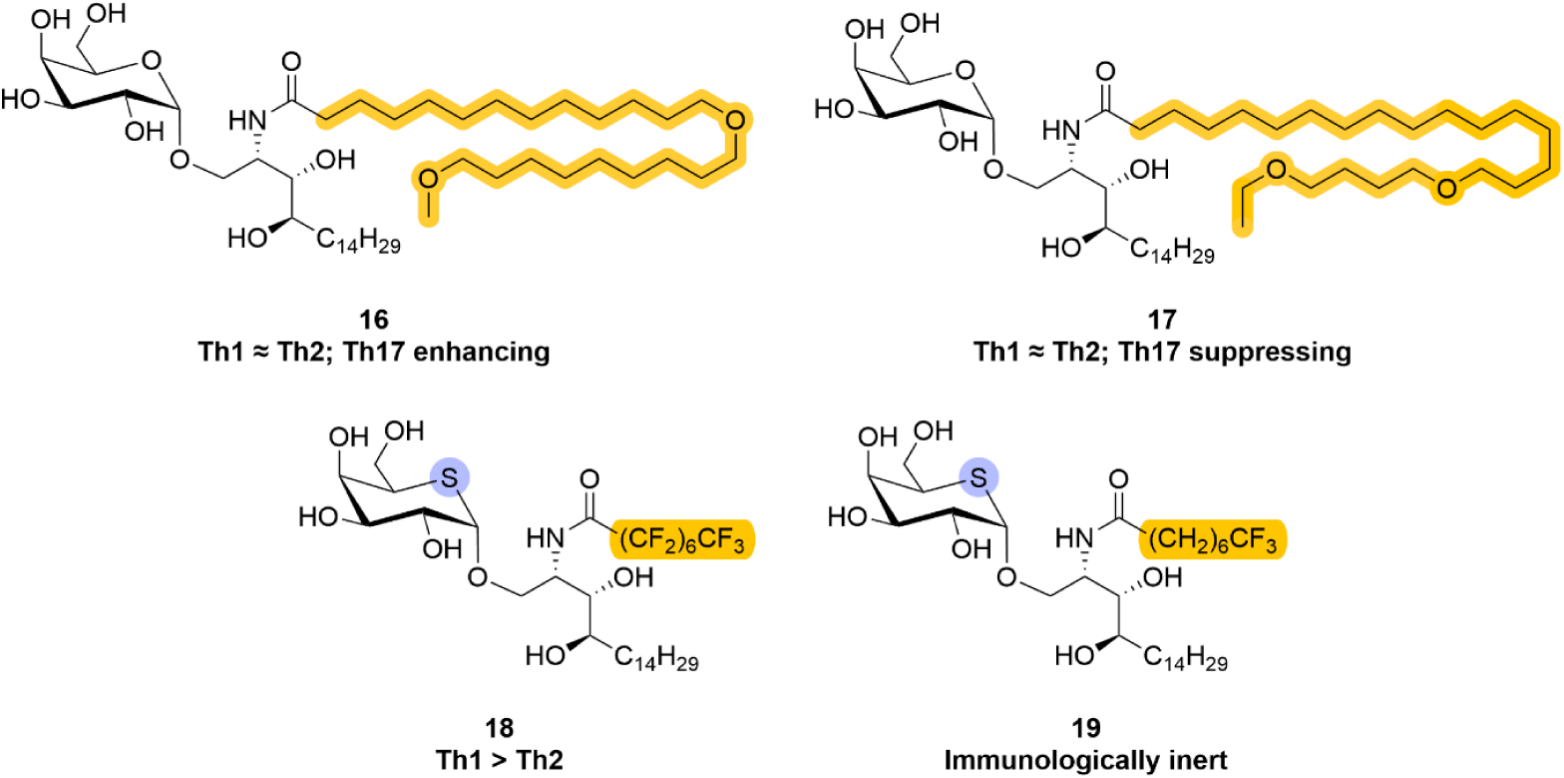
Structural modifications of the acyl chain in compounds **16**–**19** and their polarization capacity of immune response.

The integration of fluorinated lipid chains with sugar modifications has yielded even more dramatic results, exemplified by compound **18** (Figure 10) a perfluorooctanoylated 5-thio-α-GalCer analog. This compound displayed a pronounced Th1-biased immunostimulatory profile *in vivo*, inducing an exceptionally high IFN-γ/IL-4 ratio of 9:1. This enhanced activity is highly structure-specific; while full perfluorination of the octanoyl chain results in a massive boost of systemic IFN-γ, the corresponding trifluoromethylated analogs (such as compound **19**, Figure 10) remain immunologically inert. This indicates that full perfluorination is critical for the engagement of Cys12, Phe70 and Arg144 and the stabilization of the CD1d/glycolipid/TCR ternary complex, particularly when paired with a 5-thio-galactose headgroup. The combination of these two modifications appears to be synergistic, as the 5-thio-sugar derivative elicited a far more potent cytokine response than its O-glycosidic perfluorooctanoylated counterpart.^96^

The incorporation of an amide group within the acyl chain has also revealed a very interesting “anchoring pattern” in Th1/Th2 bias modulation. While it was already known that the length shortening of α-GalCer was markedly correlated with Th2-biased response, the synergy with the amide moiety promoted an even higher release of IL-4 and a decrease of IFN-γ.^97–99^ Specifically, shortening the chain to a C16-counterpart featuring an internal amide (compound **20**, Figure 11) resulted in a 2.5-fold higher selectivity for IL-4 compared to standard α-GalCer. Molecular dynamic studies revealed that these amide groups form site-specific, shielded hydrogen bonds with Ser28 and Gln14, deep within the hydrophobic A′ pocket, effectively stabilizing the complex despite the truncated acyl chain. In the same fashion, machine learning approaches and computational docking have recently been applied to predict glycolipid/CD1d/TCR interactions, guiding the design of α-GalCer-diol **21** (Figure 11). This analog, carrying two hydroxyl groups installed at positions 12 and 13 of the acyl chain, was specifically engineered to form additional hydrogen bonds with Gln14, Ser28, or Tyr73. Unlike the Th2-biasing truncated amides, α-GalCer-diol promotes a pronounced Th1-type cytokine profile with significantly higher affinity for CD1d than α-GalCer. *Ex vivo* and *in vivo* studies demonstrated that α-GalCer-diol **21** promotes a marked increase in the expansion and Th1-polarization of CD11b^+^ monocytes/macrophages, resulting in a 70% reduction in tumor nodules in B16–F10 melanoma models, making it a highly promising candidate for cancer immunotherapy.^100^

**Figure 11:**
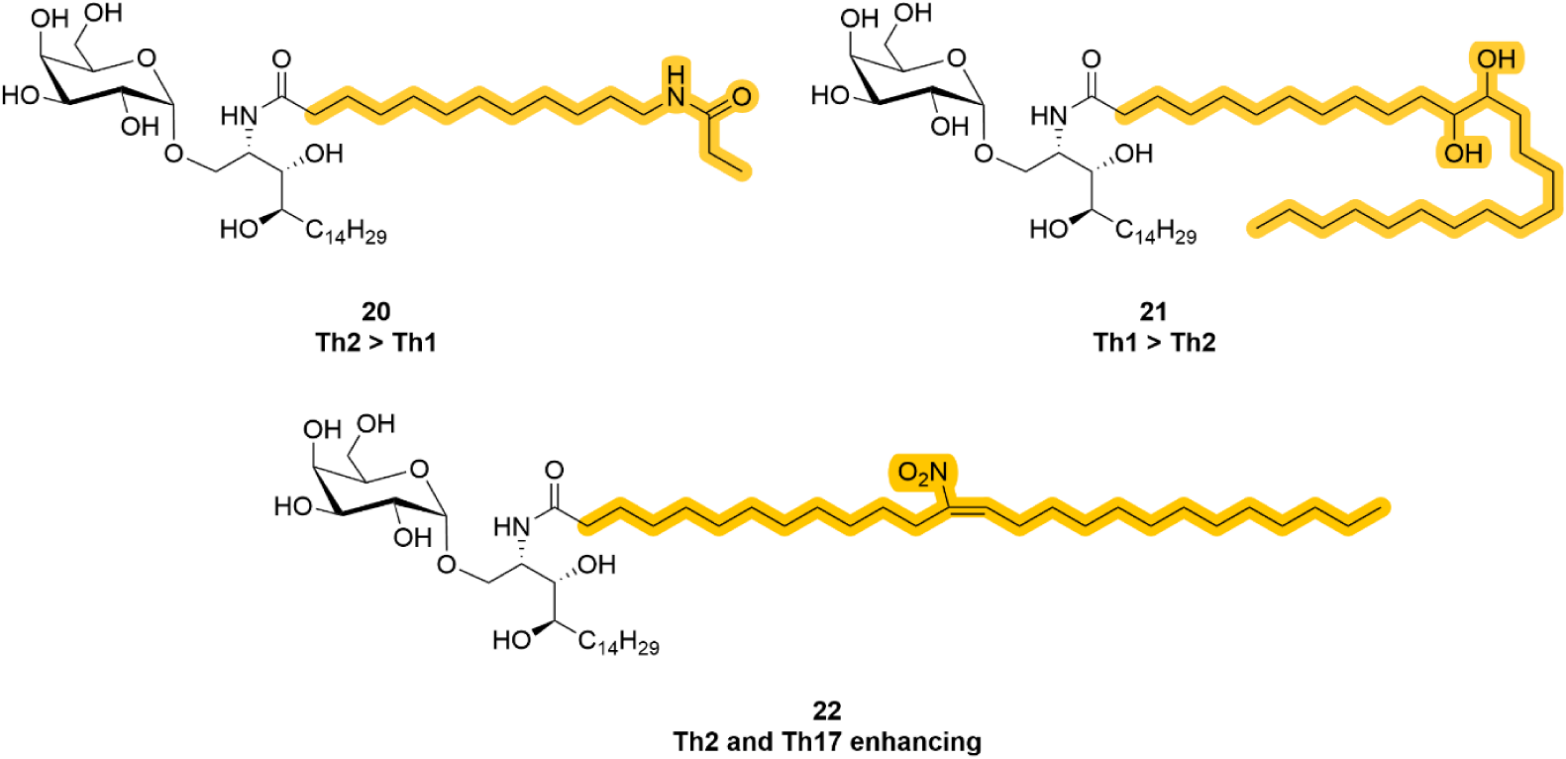
Structural modifications of the acyl chain in compounds **20**–**22** and their polarization capacity of immune response.

Nitrated acyl chain derivatives represent another class of CD1d ligands designed to modulate iNKT cell responses by incorporating a nature-inspired nitroalkene moiety. The immunological profile of these analogs is characterized by a highly selective induction of Th2 (IL-4) and Th17 (IL-17) cytokines.^101^ Biological assays and molecular dynamics simulations indicate that this distinctive selectivity is driven by stable complex formation, facilitated by hydrogen bonding with Ser28 and specialized nitro-π interactions with Phe70. While the electrophilic nitroalkene was initially hypothesized to form a covalent bond with the Cys12 residue of CD1d, similar to previously reported chloroacetyl-featuring covalent ligands (compound **7**, Figure 6), direct evidence of this covalent linkage was not observed in mass spectrometry studies. Unlike traditional Th2-biasing analogs such as OCH, which usually have reduced potency due to their shortened lipid chains, these nitrated derivatives have an exceptionally high binding affinity for CD1d, even when the chain length is the standard C26 (compound **22**, Figure 11).

Finally, the incorporation of sulfonamide functionalities has yielded some of the most potent polarization-biased agonists reported to date. The immunological phenotype of these ligands is governed by two primary parameters: (i) productive engagement of the sulfonamide with polar residues within the hydrophobic A′ pocket, notably Ser28 and Gln14, thus applying the same logic as seen with the α-GalCer-diol derivative (*vide supra*), and (ii) the overall acyl chain length. Within long-chain (C26) scaffolds, repositioning the sulfonamide by a single methylene unit toward the glycosyl headgroup is sufficient to markedly alter cytokine bias; for example, GCS-11 (compound **23**, Figure 12), bearing the sulfonamide at the 12th/13th position, induces an approximately 6-fold enhancement in IFN-γ secretion relative to α-GalCer.^102^ By contrast, progressive shortening of the lipid chain functions as a secondary structural switch that overrides sulfonamide positional effects and shifts the response toward Th2 polarization. This behavior has been attributed to altered CD1d loading kinetics at the cell surface: shortened analogs such as GCS-12–6 undergo rapid loading, favoring an early IL-4 burst, whereas sustained IFN-γ production appears to require more stable CD1d occupancy and prolonged TCR engagement. An example is C20-shortened sulfonamide GCS-12–6 (compound **24,** Figure 12), which elicits a 6.7-fold increase in IL-4 and a remarkable 76-fold enhancement in Th2 selectivity. It has also demonstrated significant protective effects against inflammatory bowel disease (IBD) in colitis models by recruiting regulatory iNKT cells to the colonic tissue.^103^

**Figure 12:**
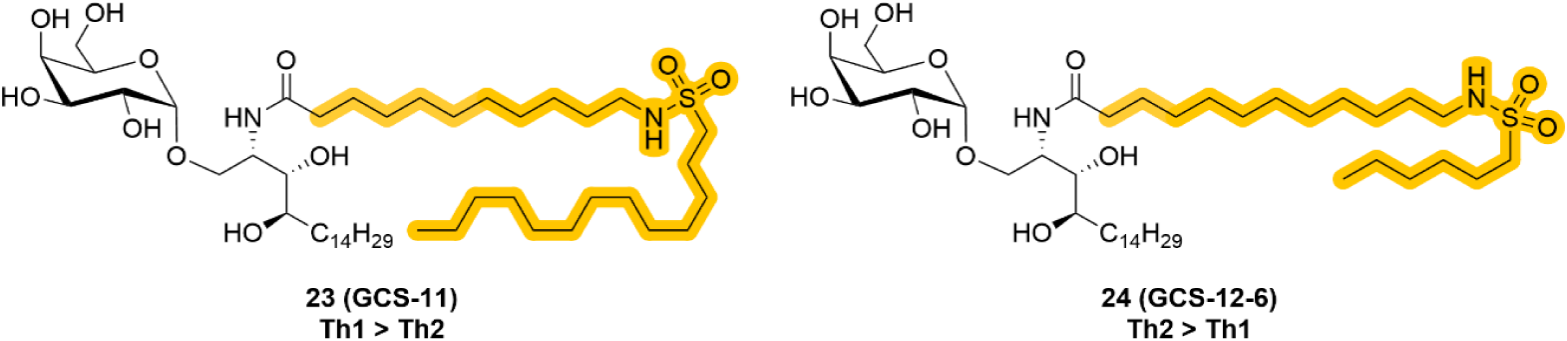
Structural modifications of the acyl chain in compounds **23** and **24** and their polarization capacity of immune response.

#### 3.1.7 Tail-End Modification of the Acyl Chain

Modulating the electronic and steric properties of the acyl terminus has proven to be one of the most effective strategies for biasing cytokine profiles while preserving the essential hydrophobic interactions required for CD1d binding. A specialized approach in this category involves the introduction of a terminal α-fluorocarbonyl moiety (exemplified by compound **25** in Figure 13). Originally designed as mildly reactive electrophiles to target the nucleophilic Cys12 residue located at the base of the A′ pocket, these analogs were intended to form covalent bonds to increase residence time. While SDS-PAGE and MALDI-TOF analyses failed to identify covalent adducts, the ligands consistently induced a high Th2-biased response. *In silico* docking suggested that the α-fluorocarbonyl group acts as a dual hydrogen-bond acceptor, forming noncovalent interactions with Gln14 and Ser28 that stabilize a unique loading orientation.^104^

**Figure 13:**
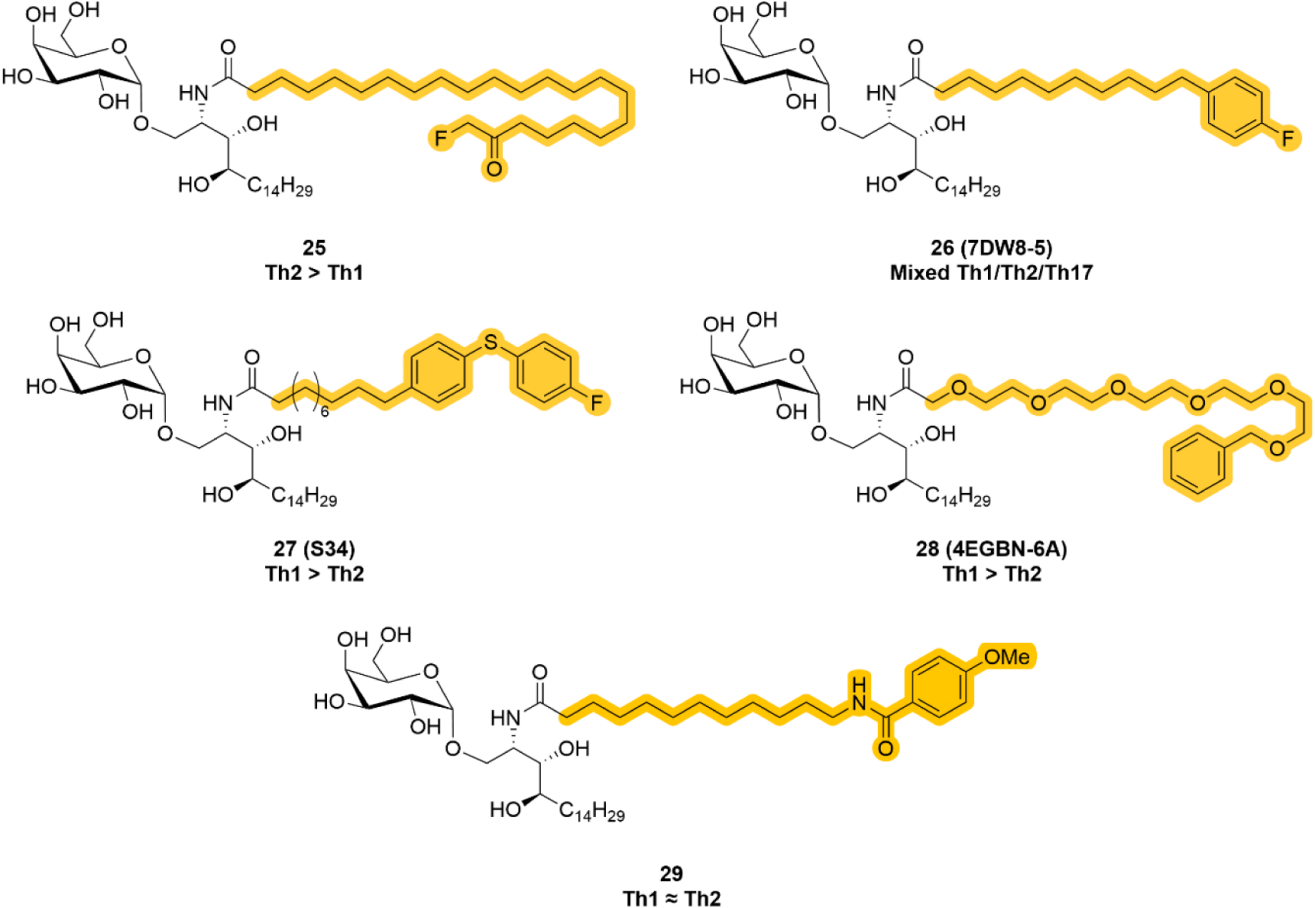
Structural modifications of the acyl chain in compounds **25–29** and their polarization capacity of immune response.

Conversely, aromatic modifications at the acyl terminus have yielded some of the most potent Th1 agonists identified to date. The most prominent example is 7DW8-5 (compound **26**, Figure 13), which features a fluorinated benzene ring on a truncated C11 acyl chain.^80^ These aromatic ligands exhibit binding avidities to human and mouse CD1d that are roughly 20-fold greater than α-GalCer, resulting in a remarkable 100-fold higher dose–effect response in stimulating human iNKT cells, with strong evidence for its protective effect against colonic inflammation in IBD.^105^ These aromatic ligands typically demonstrate the highest binding affinities for both human and mouse CD1d, as π–π stacking interactions with aromatic residues in the binding groove (specifically Tyr73 and Trp40) provide significant stabilization within the cleft.^106^

The synergy of various acyl modifications has led to the development of superior adjuvants, including thiophenyl-containing derivative S34 (compound **27**, Figure 13)^89^ and PEGylated phenyl derivative 4EGBN-6A (compound **28**, Figure 13),^107^ which exhibit remarkable dose–effect response and enhanced Th1 stimulatory activity in human iNKT cells, with the latter being able to elicit also substantial antibody titers and enhanced CTL activation. These heteroaromatic analogs induce higher IFN-γ/IL-4 ratios than their carbon-only counterparts, supporting the model that aromatic interactions stabilize the CD1d/ligand/TCR ternary complex and prolong activation.

Finally, benzamide-functionalized aromatic acyl chains offer a unique dual-mode versatility by simultaneously engaging both the hydrophilic and hydrophobic hotspots of the CD1d pocket. Molecular dynamics simulations of *p*-methoxybenzamide derivatives (compound **29**, Figure 13) show that the internal amide group forms hydrogen bonds with Ser28 and Gln14, while the terminal phenyl ring establishes π–π interactions with Phe70. This synergistic binding mode creates highly tunable modulators, where *p*-methoxy substitutions can boost cytokine induction to levels equal to or exceeding those of α-GalCer.^108^

### 3.2 Phytosphingosine Chain Modifications

#### 3.2.1 Sphinganine Chain Length

The length of the sphinganine chain (regardless of the presence of the 4′–OH) emerges as the primary structural determinant controlling TCR binding affinity to CD1d/glycolipid complexes. Truncation of this chain prevents the full occupation of the CD1d F′ channel, which accommodates alkyl chains up to 18 carbons. Truncated analog **30** (OCH, Figure 14) is known to exhibit markedly reduced TCR binding affinity (*K*_d_ = 122 μM) compared to α-GalCer (*K*_d_ = 1.6 μM), resulting in weakened immunological synapse formation, diminished calcium signaling, and impaired granule polarization in iNKT cells.^69,79,80^ Molecular dynamics simulations demonstrate that under-occupancy of the sphinganine binding pocket induces a downward settling of the Phe84 residue into the empty groove of CD1d α1 helix, causing a distortion that is allosterically transmitted to the F′ roof (Trp140, Trp153, Trp160), which alters the positioning of residues critical for TCR recognition (Glu83, Lys86, Arg89), directly correlating with reduced binding affinity and compromised iNKT cell activation.

**Figure 14:**
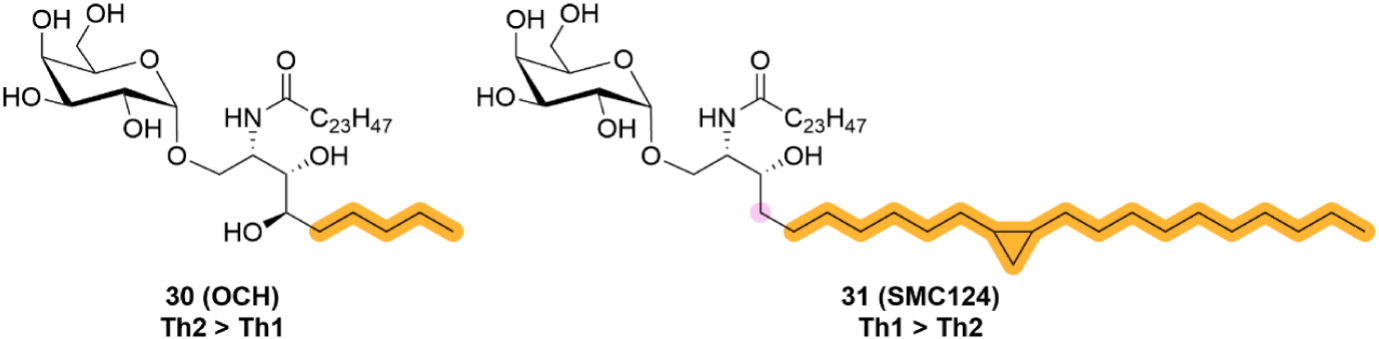
Structural modifications of the sphinganine chain in compounds **30** and **31** as well as their polarization capacity of immune response.

An interesting optimization of the sphinganine chain was achieved with compound **31** (SMC124, Figure 14), characterized by longer cyclopropane-featuring chain and a lack of C4′ OH.^109^ This derivative retains the ability to strongly activate human iNKT cells and exhibits prolonged biological stability *in vivo*. This results in sustained, high levels of systemic IFN-γ (up to seven- to 8-fold higher than α-GalCer after 22 h) due to the enhanced trans-activation of NK cells mediated by increased IL-12 secretion from DCs. X-ray crystallographic analysis suggests that these results could be explained by increased buried surface area, forcing the structure into a compressed, more compact conformation that sits deeper in the CD1d F′ pocket and engagement with Trp133.

#### 3.2.2 Alteration of the Phytosphingosine Chain

Comparative studies of phytosphingosine aminodiol analogs (lacking the 3′–OH or 4′–OH group) versus canonical aminotriols reveal that removal of one of them substantially diminishes activation of iNKT cells.^21,65,110,111^ Notably, when alternative stabilizing forces are introduced, this deficit can be largely offset by installing a covalent binder on the acyl chain,^112^ or a dihydrocinnamoyl ester at C6″ of the sugar moiety as showcased in compound **32** (AH10-7, Figure 15) with Th1-biased cytokine responses.^113^ These findings indicate that the removal of one hydroxy group does not completely abolish the productive CD1d interactions, while the removal of both leads to a complete loss of activity.^65^ α-GalCer analogs incorporating terminal iso-branched sphinganine backbones in concert with a chloroacetamide moiety on the acyl chain have been reported as possible solution to bypass this requirement, as in compound **33** (Figure 15), eliciting strong iNKT cell activation accompanied by a pronounced Th2-biased cytokine response.^112,114^ It is worth noting that this structural strategy finds a distinct natural parallel in the *Bacteroides fragilis*-α-galactosylceramides constitutively produced by the human gut symbiont *B. fragilis*, which stimulate iNKT cells to produce a distinct immunoregulatory shift, favoring the secretion of the anti-inflammatory cytokines IL-10 and IL-13 while reducing levels of the pro-inflammatory IFN-γ.^115,116^

**Figure 15:**
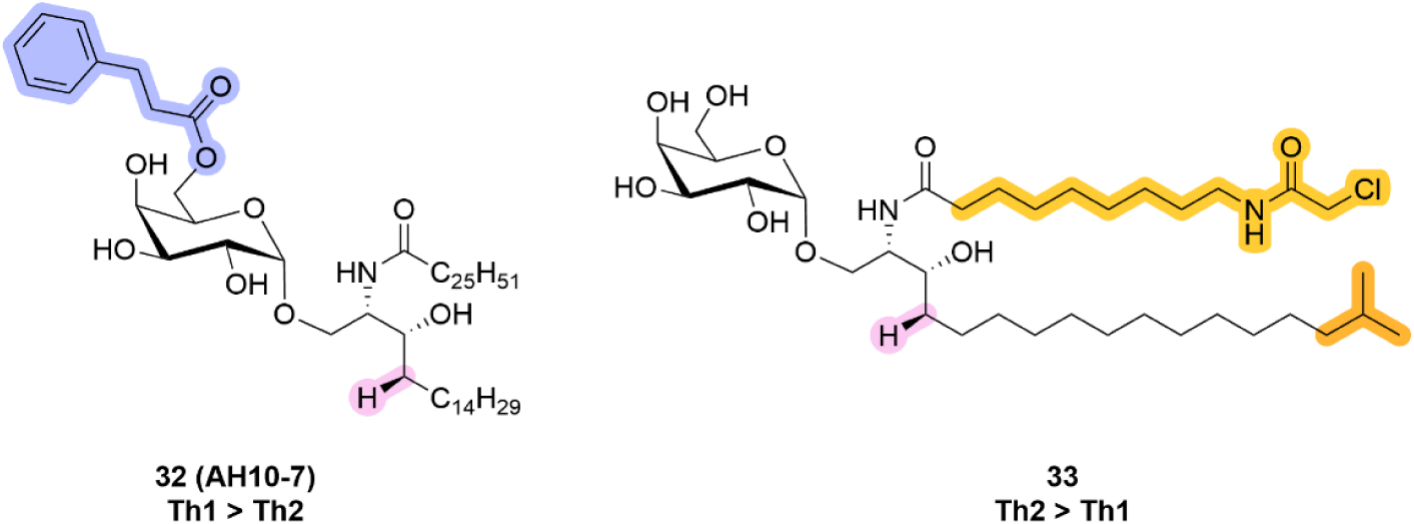
Structural modifications in compounds **32** and **33** as well as their polarization capacity of immune response.

#### 3.2.3 Heteroatom-Containing Derivatives and Heteroaromatic Modifications

α-Galactosylsphingamide **34** (Figure 16) was synthesized based on evidence that aromatic modifications such as terminal phenyl and pyrazolyl groups selectively stimulate iNKT cells with enhanced Th2-type cytokine secretion.^117^ While it maintained the TCR binding affinity *in vitro*, it exhibited poor *in vivo* activity due to compromised antigen presentation. The crystal structure analysis revealed that while the introduction of an amide group could interact with Tyr73, this modification resulted in altered CD1d conformation, thus highlighting inflexibility to polar modifications and the strict requirement of the hydrophobic nature of the F′ channel during rational design.^118^ On the other hand, the substitution of the amide moiety with a pyrazole as seen with compound **35** (Figure 16) exhibited greater selectivity toward secretion of the immunomodulatory cytokine IL-4 both *in vitro* and *in vivo*, suggesting that the F′ channel can accommodate heteroaromatic modifications to establish π–π stacking interactions with Phe84, Trp133 and Phe77 of the F′ roof.^117,119^ Compound **35** was also evaluated for experimental autoimmune encephalitis (EAE), where a single dose dramatically ameliorated EAE, compared to α-GalCer-treated animals.

**Figure 16:**
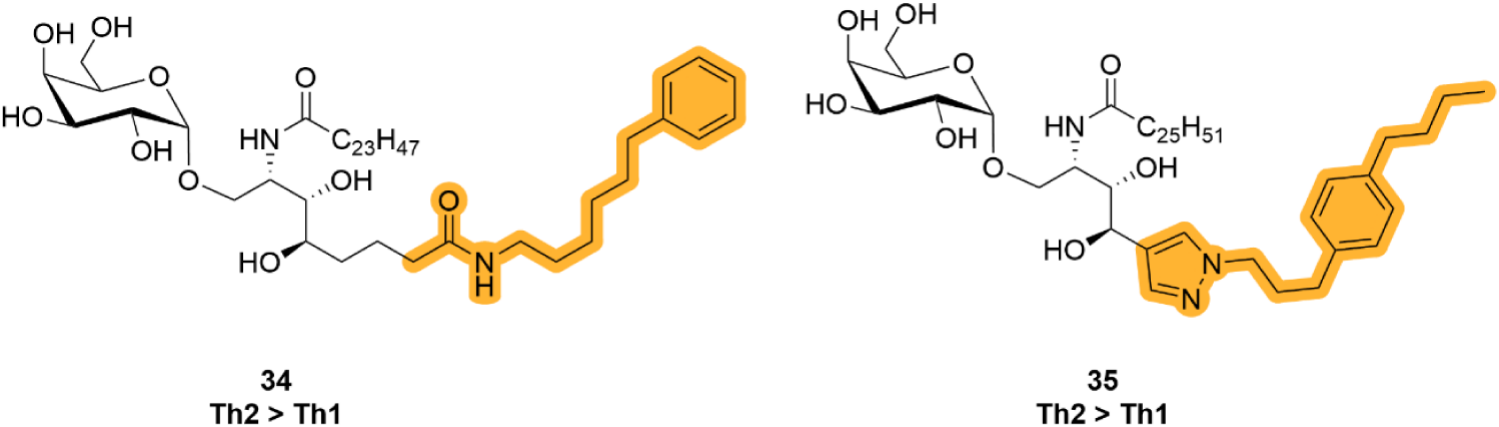
Structural modifications of the sphinganine chain in compounds **34** and **35** as well as their polarization capacity of immune response.

### 3.3 Sugar Headgroup Modifications

#### 3.3.1 Glycosidic Linkage Modifications

The α-anomeric configuration at the C1″ position of the galactosyl headgroup of α-GalCer is the favorite, but not exclusive,^8,120^ configuration for recognition by iNKT cells and CD1d binding. iNKT TCRs can recognize β-linked self-antigens like iGb3 or β-ManCer through induced-fit molecular mimicry, where the TCR flattens the headgroup to resemble an α-linked orientation.^121,122^ It should be noted that β-GalCer is a very weak iNKT-cell agonist; it required a 3 orders of magnitude higher dose to achieve the same level of efficacy (i.e., tumor protection) as α-GalCer.^123^

The most significant structural modifications at the C1″ position do not concern the spatial orientation of the glycosidic bond, but rather its atomic composition. For instance, by replacing the C1″ oxygen with a methylene group, the α-C-galactosyl analog (α-C-GalCer, compound **36**, Figure 17) was specifically designed to overcome the inherent hydrolytic instability of glycosidic bonds to enzymatic degradation. This enhanced stability drives an enhanced Th1-biased response, yielding as much as a 1000-fold higher protection against malaria and melanoma metastasis in mice compared to α-GalCer.^124^ Despite its promising results in murine models, α-C-GalCer displayed a notable species-specific limitation, acting only as a weak agonist for human iNKT cells.^125^ This has been circumvented by the development of restricted (*E*)-α-C-GalCer (GCK152, compound **37**, Figure 17), which incorporates an *E*-alkene linker. This geometry is hypothesized to fit more precisely into the CD1d groove, restoring potent stimulatory activity in human PBMCs.^126,127^ α-S-GalCer (compound **38**, Figure 17) has also been explored; it offers improved solubility and stability in biological systems while retaining the ability to induce cytokine release, promote DC maturation, and support iNKT cell-mediated responses, although its overall potency is comparable to or slightly lower than that of α-GalCer.^128^

**Figure 17:**
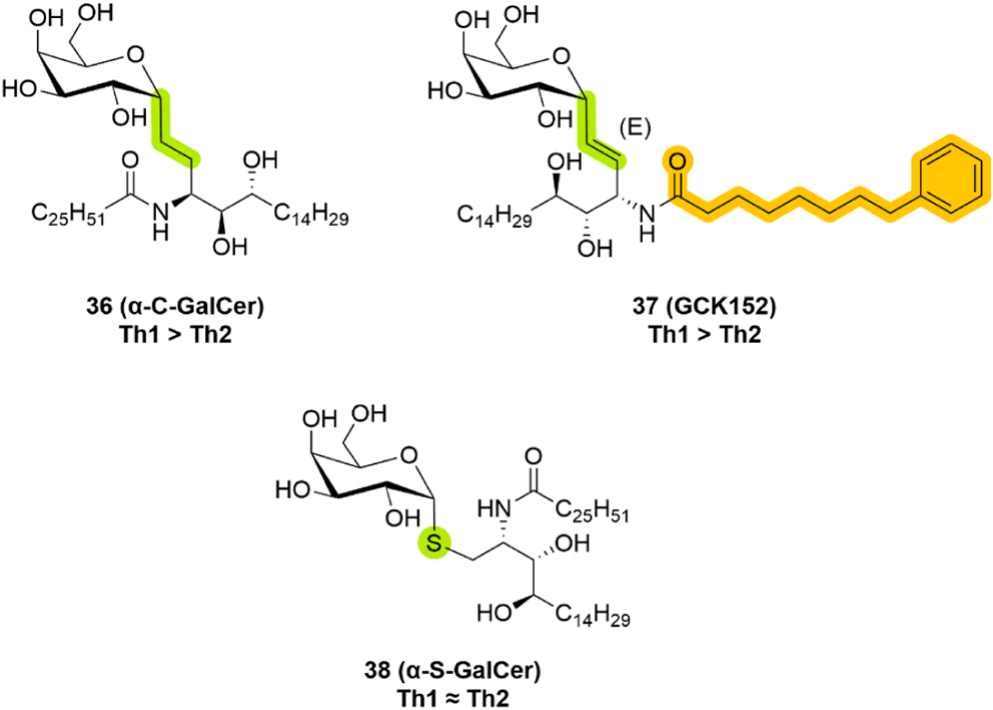
Structural modifications in compounds **36**–**38** and their polarization capacity of immune response.

#### 3.3.2 C2″ Galactose Ring Modification

Both removal and substitution of the C2″ hydroxyl group abolishes iNKT cell agonist activity. For example, the replacement of the hydroxyl group by an amine resulted in inactive compound **39** (Figure 18).^21^ Similarly, the study of 2-exomethylene pseudoglucosylceramides (represented by compound **40**, Figure 18), glucose derivatives of α-GalCer lacking the C2″–OH group entirely as the sp3-hybridized C2″ position is converted into an sp2-hybridized exomethylene group further exposed this structural requirement. Biological evaluations demonstrate that neither the pseudo-α-GC nor the pseudo-β-GC variants are capable of activating the iNKT cell population. Interestingly, while this C2″ modification terminates CD1d binding, it simultaneously confers a novel functional profile upon the glycolipid scaffold by enabling interaction with different lectin receptors.^129^ Specifically, pseudo-α-glucosylceramides selectively engage the macrophage-inducible C-type lectin (Mincle), whereas the corresponding native glucosylceramides elicit negligible signaling. This modification thus serves as a molecular switch that diverts recognition from the CD1d/iNKT axis to Mincle, conferring distinct receptor selectivity.^130^ The total loss of CD1d-mediated iNKT activity in these analogs reflects the disruption of a critical hydrogen-bonding network involving the C2″–OH and residues Asp151 on CD1d, as well as Gly96 on the iNKT TCR, underscoring the non-negotiable importance of this position for CD1d recognition.

**Figure 18:**
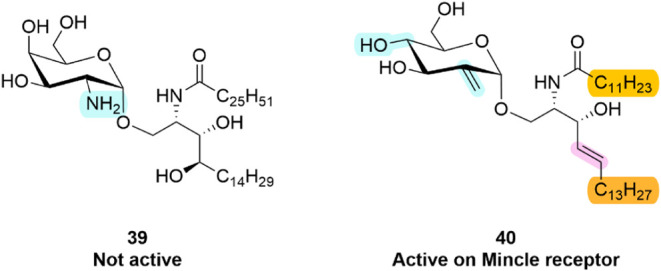
Structural modifications in compounds **39** and **40**.

#### 3.3.3 C3″ Galactose Ring Modification

Mutational analysis has demonstrated that the 3″–OH group is essential for efficient recognition by the iNKT cell TCR and upon removal or alteration usually lead to loss in activity, with a few exceptions (3″-deoxy and 3″-fluoro analogs) with reduced antigenic activity.^131,132^ It has been observed that 3″-sulfo-β-galactosyl ceramides with sphingosine (Δ4,5 unsaturation) chain like C24:2-sulfatide (compound **41**, Figure 19) are able to activate iNKT cells and possess potent IFN-γ-dependent antitumor effects *in vivo.* ^133,134^ Crystal structure analysis revealed that the β-anomeric linkage of sulfatide causes the 3″-sulfated galactose headgroup to project perpendicularly upward, away from the CD1d binding pocket. Within this complex, the sulfate group remains only loosely bound to CD1d and is rapidly exchanged or repositioned during the interaction with the TCR. Nevertheless, the stimulatory activity of these ligands does not seem to be a direct effect of the intact sulfatide, but it is driven by lysosomal processing within DCs, where the enzyme arylsulfatase A cleaves the sulfate moiety to generate β-GalCer. This processed form, in turn, changes the specific immune recognition from type II NKT to iNKT cells through the mechanism of induced-fit molecular mimicry, wherein the TCR flattens the perpendicularly oriented β-sugar to resemble the prototypical α-linked orientation. Interestingly, altering the anomeric configuration results in loss of activity for the 3″-sulfated α-galactosylceramide. This suggests that sulfatide-reactive NKT cells are selective for the configuration of the sulfated galactose moiety, with the β form being active and the α form being inactive. By contrast, derivatives with a phytosphingosine base (such as pC24:2-sulfatide, with a C4′–OH group; compound **42** in Figure 19) are less selective and are able to activate iNKT cells without lysosomal processing; however, they were found to be weaker than their sphingosine-based counterparts.

**Figure 19:**
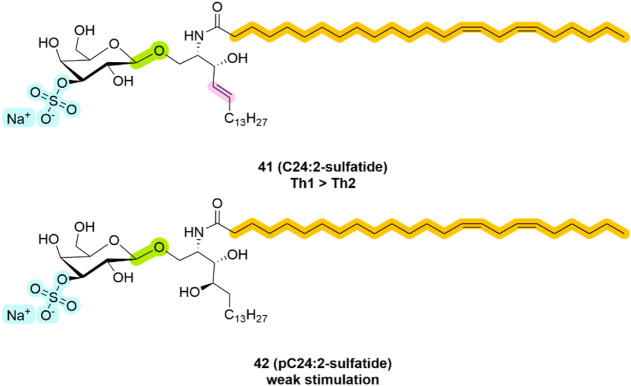
Structural modifications in compounds **41** and **42** and their polarization capacity of immunologic response.

#### 3.3.4 C4″ Galactose Ring Modification

The C4″–OH group of the galactosyl headgroup is a critical determinant of the maximal agonistic activity observed with α-GalCer, because it provides an optimal stabilizing hydrogen bond with the iNKT TCR, specifically engaging Phe29. While the C2″ and C3″ positions are strictly sensitive to modification, the C4″ position is situated in a more solvent-accessible cavity, rendering it more permissive to structural diversification. Preliminary studies demonstrated that simple removal or alteration of this group as in 4′-deoxy-α-GalCer (compound **43**) or aromatic variants such as Ar3-GSL (compound **44**) (Figure 20) generally results in a marked decrease in stimulatory potency compared to α-GalCer.^35,132,135,136^

**Figure 20:**
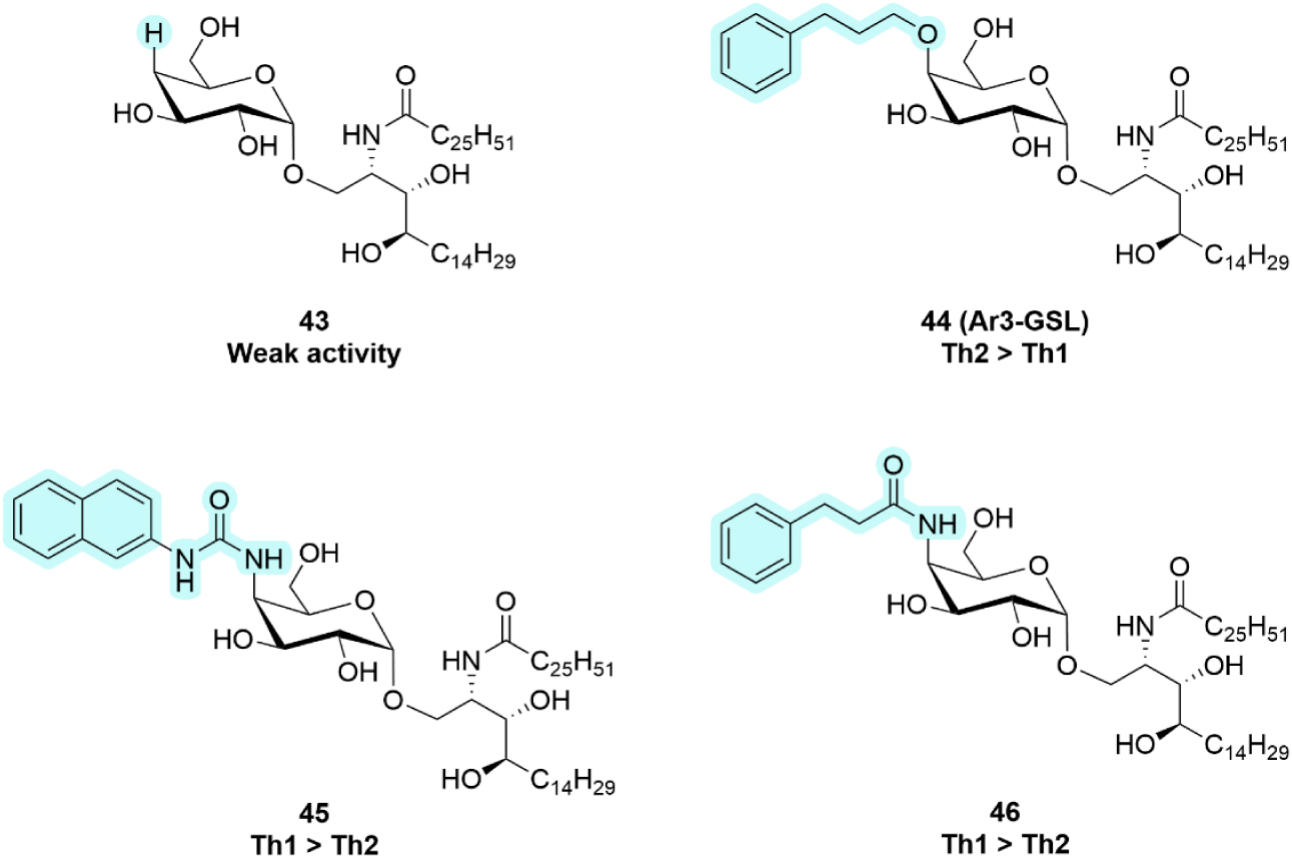
Structural modifications of the galactose head in compounds **43**–**46** and their polarization capacity of immune response.

More recently, research has shifted toward utilizing the C4″ position to introduce bulky, functionalized moieties that can establish novel interactions within the CD1d/TCR interface. Notable examples include variants incorporating a naphthylurea moiety (compound **45**, Figure 20) or amide-linked phenyl alkane substitutions (compound **46**, Figure 20).^137^ These compounds are particularly noteworthy because, while they may show weak activity in mouse models, they exhibit substantially greater agonistic activity for human iNKT cells and induce potent antitumor immunity in humanized mouse models, making them promising candidates for immunotherapy. The increased activity of the C4″-amide variants in humans is primarily a result of an additional interaction with the human iNKT TCR through Phe51 after the glycolipid is bound to CD1d, stabilizing the immunological synapsis and shifting the cytokine balance toward a pro-inflammatory Th1-biased response.

#### 3.3.5 Ring Oxygen Replacement Strategies

5-Thio-α-GalCer (exemplified by compound **47**, Figure 21), which features a sulfur atom substituting for the endocyclic galactopyranose ring oxygen, demonstrates significantly enhanced Th1-biased responses in both *in vitro* and *in vivo* models.^138^ The larger atomic radius of sulfur compared to oxygen alter ring geometry and hydrogen bonding patterns, contributing to the observed immunological bias. The tailored activity of sulfur-containing analogs suggests that the CD1d binding site can accommodate modest changes in the ring heteroatom, which provided a foundation for exploring electronic and steric requirements for sugar recognition and design of conformationally restricted-sp^2^-iminosugars such as compound **48** (Figure 21), featuring a bicyclic cyclic carbamate moiety.^139^ These compounds offer enhanced stability against glycosidase degradation and can act as either iNKT antagonists or mild agonists depending on their lipid tail configuration, showing promising results for treating conditions such as asthma and autoimmune hepatitis.

**Figure 21:**
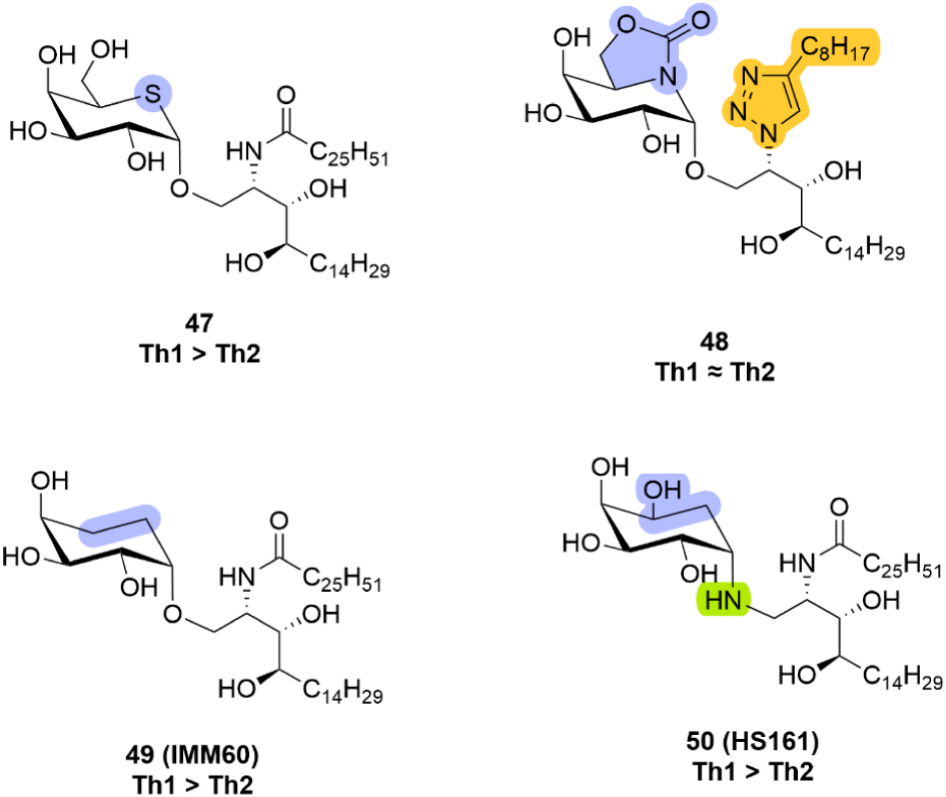
Structural modifications of the galactose head in compounds **47**–**50** and their polarization capacity of immune response.

Carbocyclic derivatives such as **49** (IMM60, Figure 21) are alternative galactopyranose modifications derived from a truncated α-GalCer scaffold and designed to reduce headgroup flexibility relative to earlier threitol analogs. This structural rigidification enhances CD1d/lipid binding affinity and prolongs iNKT/TCR interaction compared to α-GalCer (*K*_d_ (compound **49**) = 0.61 μM versus *K*_d_ (α-GalCer) = 1.12 μM). Functionally, compound **49** displays superior *in vivo* potency, inducing elevated IFN-γ secretion, higher frequencies of antigen-specific T-cell responses, and potent antitumor activity in the B16 melanoma model at lower doses than α-GalCer. These properties are likely linked to improved bioavailability rather than TCR affinity alone, but nonetheless position **49** as a promising iNKT cell agonist for clinical development as an immune adjuvant.^140–142^

The combination of different galactose modifications led to the development of several NH-aminocyclitol derivatives, where the substitution of the glycosidic bond confers complete resistance to enzymatic hydrolysis by glycosidases, thereby ensuring prolonged bioavailability and sustained iNKT cell stimulation *in vivo*. Compound **50** (HS161, Figure 21) outperforms standard α-GalCer in inducing IFN-γ while minimally eliciting Th2 cytokines, providing superior protection in tumor and asthma models without a massive cytokine storm or subsequent anergy associated with α-GalCer. Crystallographic modeling revealed that the amino-cyclitol head occupies an orientation almost identical to the parent glycolipid, maintaining the three critical hydrogen bonds with Asp80, Asp151 and Thr154 at the entrance of the binding groove, but with an additional stabilizing interaction with TCR Gly96.^143,144^

#### 3.3.6 C6″ Galactose Ring Modification

The C6″ position of the galactosyl moiety is an optimal site for chemical modification, as its primary hydroxyl group is not strictly required for CD1d binding or TCR recognition. Moreover, unlike the buried C2″ and C3″ positions, the C6″–OH is solvent-exposed and points away from the binding groove, making it highly tolerant to structural diversification.^111,145^ This position has become a key hotspot for introducing bioconjugation handles, improving solubility, and fine-tuning the iNKT cell cytokine response toward pro-inflammatory (Th1) or anti-inflammatory (Th2) profiles. A foundational advancement in this area was the development of 6″-azido-6″-deoxy-α-GalCer (compound **51**, Figure 22) by the Besra and Cox groups.^146^ This versatile precursor serves as a bio-orthogonal platform for click chemistry. While the azido precursor itself acts as a potent agonist with a slight Th1-biasing profile, its real utility lies in the possibility to convert the azido handle into amide- or triazole-containing conjugates, which will be discussed in the next chapter.

**Figure 22:**
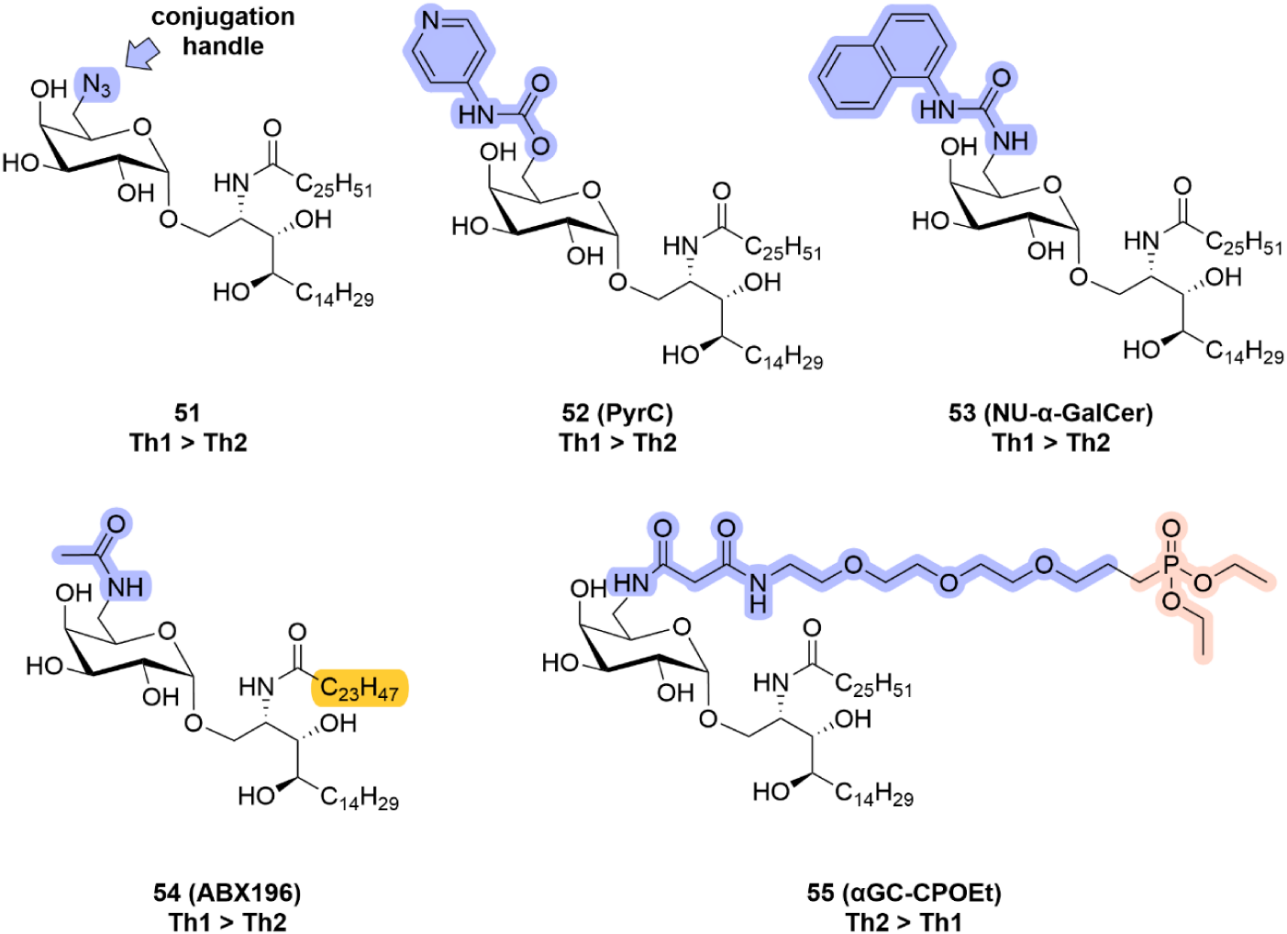
Structural modifications introduced at the C6″ position in compounds **51–55** and their polarization capacity of immune response.

The introduction of aromatic moieties at C6″ has proven even more effective for inducing marked pro-inflammatory responses. PyrC-α-GalCer (pyridin-4-yl carbamate, compound **52**, Figure 22) and NU-α-GalCer (naphthylurea derivative **53**, Figure 22) are distinguished by their superior Th1-polarizing potential and enhanced antitumor activity in murine melanoma models.^147–149^ Crystal structure analysis revealed that these aromatic groups act as additional anchors; for example, the naphthyl moiety occupies a hydrophobic pocket in CD1d created by the displacement of Met69, while the pyridine ring of PyrC establishes novel, stabilizing contacts with TCR residue Gln52. ABX196 (6″-acetamido-α-GalCer derivative **54**, Figure 22) elicited a more potent Th1-skewed response compared to α-GalCer, while maintaining an excellent safety profile in humans. It has been clinically validated as a potent vaccine adjuvant, demonstrating the ability to induce protective anti-HBs antibody responses in healthy volunteers after a single dose in Hepatitis B vaccine trials.^150–152^

More recently, C6″ modifications have expanded into modern vaccine platforms with the discovery of αGC–CPOEt (compound **55**, Figure 22), which incorporates a phosphonate diester linked via a hydrophilic PEG4 spacer. When used as an adjuvant for the SARS-CoV-2 RBD-Fc subunit vaccine, αGC–CPOEt elicited a rapid, IL-4-skewed cytokine burst that correlated with exceptionally strong humoral immunity. Remarkably, it evoked neutralizing antibody responses approximately 5.5-fold higher than those induced by α-GalCer and 25-fold higher than unadjuvanted vaccines, thus emerging as a promising candidate for future COVID-19 vaccine formulations.^153^

#### 3.3.7 Conformationally Restricted Analogs

Rational design based on crystallographic analysis of CD1d/ligand/TCR complexes has guided the development of conformationally restricted analogs that rigidify flexible galactose headgroup conformations. These include 4,6-O-galactosyl restricted analogs such as CH_2_–αGC, Me_2_C-αGC, 5mem-αGC, and 6mem-αGC, designed to lock the galactose ring into its bioactive conformations for TCR recognition.^154^ Structural studies confirm that standard α-GalCer binds with a consistent pattern where the C4″–OH is perpendicular and the C6″–OH is parallel to the galactosyl ring; by installing cyclic acetals, these analogs effectively preorganize the sugar into this conserved pattern, minimizing the entropic penalty associated with TCR binding. Variants such as SO-αGC and phenyl-modified acetals like PhC-αGC (compounds **56** and **57**, respectively, in Figure 23) have been engineered to enhance potential π–π stacking interactions with aromatic residues in the CD1d/TCR interface. Functionally, these conformationally restricted scaffolds have proven to be exceptionally potent vaccine adjuvants. In SARS-CoV-2 RBD-Fc subunit vaccination models, mice immunized with these analogs produced pronounced anti-RBD antibody responses and neutralizing titers that were statistically comparable to α-GalCer.^155^

**Figure 23:**
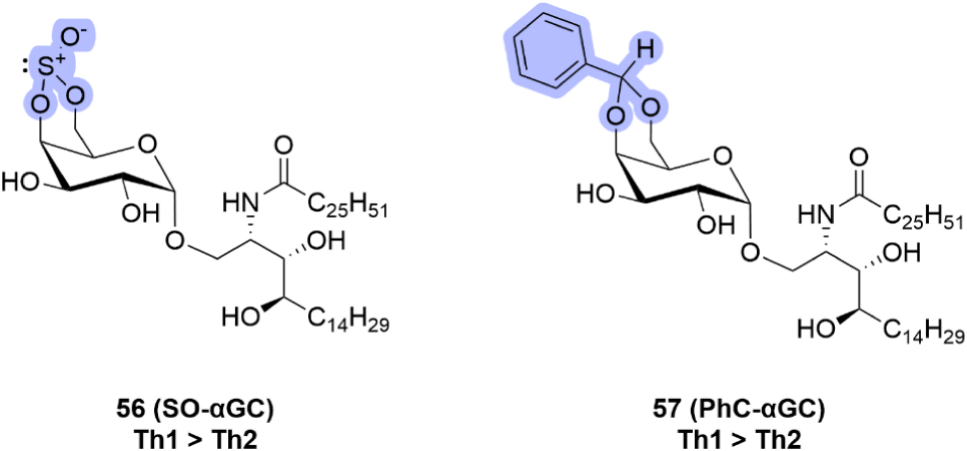
Structural modifications in compounds **56** and **57** as well as their polarization capacity of immune response.

#### 3.3.8 Alternative Glycosidic and Non-Glycosidic Chemotypes

α-Glucosylceramide (α-GlcCer, compound **58**, Figure 24) is a naturally occurring mammalian glycosphingolipid that is constitutively produced by immune cells. It functions as a dominant endogenous CD1d ligand and plays a critical role in the thymic selection of iNKT cells through CD1d-mediated antigen presentation.^12,156^ In this context, α-GlcCer appears to be more potent than the prototypical α-GalCer, which instead serves primarily as a strong activating ligand for peripheral iNKT cells when presented by DCs. β-Mannosylceramide (β-ManCer, compound **59**, Figure 24) represents a conceptual departure from the classical α-linked CD1d ligands, demonstrating that β-linked glycolipids can also drive robust immune activation.^121^ Unlike α-GalCer, which mediates tumor regression via an IFN-γ-dependent mechanism, β-ManCer induces antitumor immunity that is largely independent of IFN-γ but strictly dependent on nitric oxide synthase (NOS) and TNF-α.^157^ Nonetheless, β-ManCer proved to be a potent agonist, capable of inducing strong tumor protection even at a low dose (approximately 10-fold lower potency than that of α-GalCer), whereas α-fucosylceramide (α-FucCer) demonstrated no antitumor activity in this model, and it failed to induce any tumor protection.^123^ When administered concomitantly at doses too low for either to protect alone, β-ManCer and α-GalCer successfully protected mice against tumors, suggesting potential synergism. Remarkably, anti-CD1d/α-GalCer monoclonal antibodies retain the capacity to detect CD1d-bound β-ManCer despite its β-glycosidic linkage, underscoring the structural and antigenic overlap between these distinct classes of CD1d ligands.

**Figure 24:**
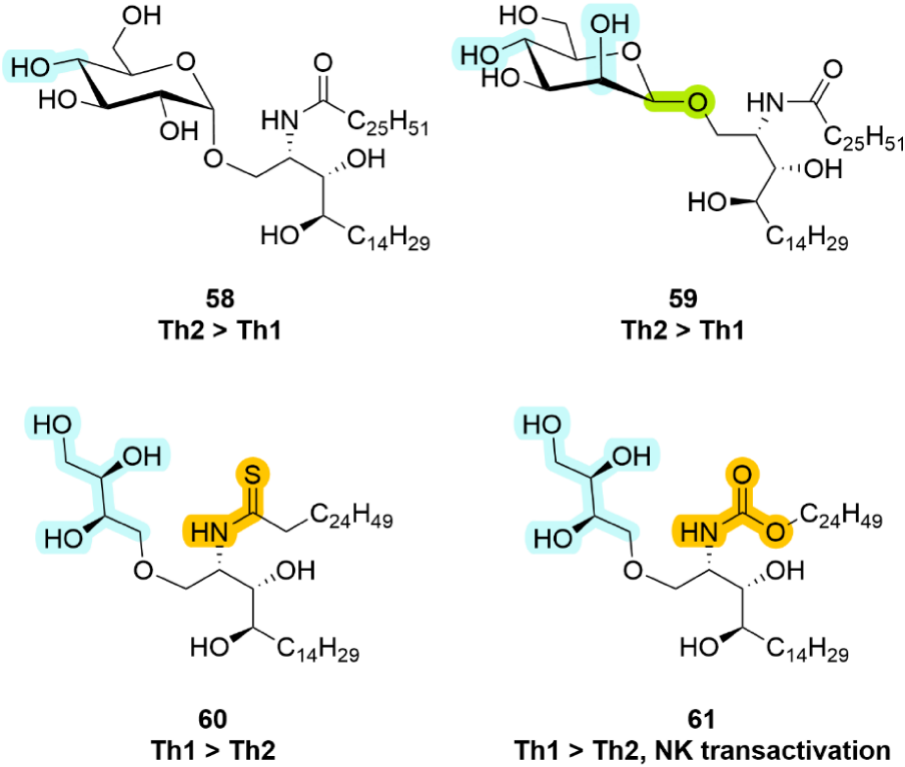
Structural modifications in compounds **58**–**61** and their polarization capacity of immune response.

The development of sugar-alcohol derivatives such as glycerolceramide, threitolceramide (ThrCer), and arabinitolceramide, incorporating three, four, and five hydroxymethylene groups, respectively, represents a significant deviation from traditional glycosidic ligands. These mimetics offer advantages such as simplified chemical synthesis, enhanced biological stability, and reduced immunogenicity compared to complex glycolipids. However, their acyclic nature results in higher conformational flexibility compared to the rigid pyranose sugar of α-GalCer, contributing to their status as weaker iNKT cell agonists. Nonetheless, a major advantage of the ThrCer scaffold is that it avoids the long-term functional anergy associated with α-GalCer, allowing iNKT cells to recover their ability to produce cytokines within 14 days of administration.^158^ Building upon this, researchers have produced strongly Th1-polarized cytokine profiles by combining the acyclic scaffold with bioisosteric amide replacements, mainly ThrCer thioamide (compound **60**, Figure 24) and carbamate (compound **61**, Figure 24) derivatives.^87^ Thioamide derivatives are better hydrogen-bond donors than classical amides due to increased polarity and N–H acidity, and induce significantly higher activation and ensuing IFN-γ production than the parent ThrCer alcohol, while producing no detectable IL-4 burst at 2 h. Carbamate derivative also induces increased iNKT activation and maintains a distinct Th1 bias. It has the added benefit of transactivating NK cells to sustain IFN-γ production. Furthermore, carbamate derivatives are significantly shorter to synthesize than their urea counterparts, making them more attractive for clinical development.

Finally, nonglycosidic chemotypes such as compound MCS-0208 (2-(hydroxymethyl)­phenylthio-phytoceramide, compound **62**, Figure 25) demonstrate how a simple aromatic ring can serve as a functional surrogate for the sugar headgroup typically required for activity. In biological assays, MCS-0208 has shown human iNKT cell activation despite lacking the galactose 2″–OH and 3″–OH groups, as the 2-hydroxymethyl group was able to mimic the bond to Asp153 on the CD1d α1 helix.^159^ The potency of this aryl-ceramide family is highly dependent on the acyl chain length; analogs with shorter acyl chains (compound **63**, Figure 25) exhibit significantly reduced activity compared to the C26 chain of MCS-0208. This activity is explained by its unique binding mode which allow a π–π stacking interaction between the phenyl ring and Trp153 of the CD1d α2 helix.

**Figure 25:**
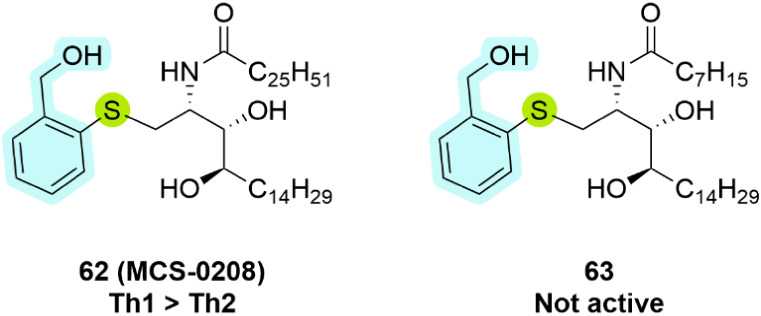
Structural modifications in compounds **62** and **63** and their polarization capacity of immune response.

### 3.4 Summary of SARs

It has been widely recognized that the antigenic potency of α-GalCer analogs is affected by structural modifications that stabilize the CD1d/glycolipid complex and optimize T-cell receptor engagement.^67,96,148,149^ A consistent finding across many α-GalCer analogs is that long-lived, high-avidity CD1d/glycolipid/TCR complexes consistently correlate with stronger, sustained Th1 responses and superior antitumor efficacy, whereas less stable, rapidly displaced or surface-loaded complexes favor Th2-biased or mixed profiles.^160–163^

While long, saturated acyl chains (C24–C26) provide a strong foundation for activity, higher potency than that of α-GalCer can be achieved by engineering the acyl chain to stabilize its fit within the hydrophobic A′ pocket. Introducing terminal phenyl or *para*-fluorophenyl motifs, particularly on shorter chains, increases hydrophobic contacts and stabilizes the A′ pocket, which provides higher release of IL-2 in cytokine assays. Incorporating polar elements, such as amides, benzamides, sulfonamides and polyethylene glycol motifs into the acyl chain creates additional hydrogen-bonding networks with buried residues inside the CD1d cavity (particularly Ser28 and Gln14). These extra noncovalent interactions yield top-tier agonists with very high cytokine output by promoting longer-lived CD1d/glycolipid complexes *in vivo*. Polarization is then tuned by these modifications: aromatic motifs at the tail-end or conformational restriction through branching typically shift the response toward a Th1 profile, whereas shortening the fatty acid chain or introducing unsaturation skews the response toward Th2 (Figure 26). The internal architecture of the lipid tail is also critical. Modern designs exploit branched motifs that clasp around the hydrophobic poles of the CD1d binding pocket, restricting torsional flexibility and thereby reducing the entropic penalty of binding, which translates directly into higher potency and Th1 skewing. It is worth noting that specialized responses involving IL-17 have also been observed with nitrated acyl chain derivatives and specific diether modifications.

**Figure 26:**
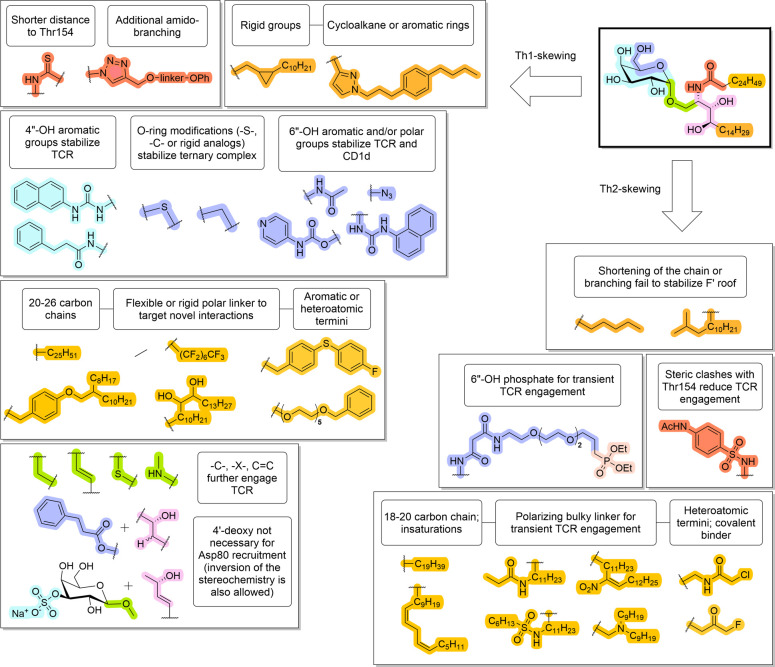
Updated SAR overview of α-GalCer analogs with structural modifications responsible for Th1- or Th2 polarization of subsequent immune response. Figure was created using ChemDraw.

As with the phytosphingosine chain, its analogs primarily bias cytokine profiles, as seen with shortened chain length; further changes to this chain significantly alter CD1d complex stability and can easily disrupt the ternary complex. Nevertheless, correctly positioning the 2′–NH and 3′–OH groups on the phytosphingosine chain is essential for optimal glycolipid orientation within CD1d; loss or shift of these groups reduces antigenic strength. Bioisosteric replacement with thioamides or carbamates, can enhance hydrogen-bonding or contact with CD1d Thr154, skewing the polarization toward Th1 profile. Unmodified galactose stereochemistry is optimal for activating invariant NKT cells, and precise derivatizations that preserve the critical TCR-facing geometry can augment both potency and Th1 polarization. Specifically, modifications at the C6″ and C4″ positions with hydrophobic, aromatic, or naphthylureido groups can yield strong stimulation by forming unique noncovalent interactions with additional CD1d amino acids (like Thr159) or by increasing the half-life of the CD1d/glycolipid/TCR trimolecular complex (stabilizing Phe29 and Ser30), which directly correlates with amplified overall immunological potency and a strong Th1 bias.

It is interesting to note that, even when TCR recognition remains unaltered, lipid structural variations can selectively activate distinct Th1 or Th2 cellular networks. This polarization is thought to be shaped by membrane raft localization and the specific APC subsets engaged *in vivo*, observations that together suggest the Th1/Th2 bias of iNKT responses cannot be fully accounted for by ternary complex stability alone.^164,165^ Rather, structural determinants are proposed to act in concert with the CD1d loading route, APC subset identity, and raft localization to collectively govern complex avidity, stability, and conformational dynamics at the TCR interface, as well as the duration and cellular context of downstream signaling.

A key complementary mechanism governing Th1/Th2 bias is the spatial distribution of CD1d-lipid complexes on the cell surface.^166–168^ Strong Th1-biasing agonists require endosomal loading via lipid transfer proteins and subsequently localize preferentially within detergent-resistant lipid raft microdomains, an association that is essential for robust TCR signal transduction, IFN-γ production, and the secondary recruitment of NK cells. In contrast, Th2-biasing variants bearing short or unsaturated lipid chains (such as α-GalCer C20:2) exhibit higher aqueous solubility, enabling direct surface loading onto CD1d, and are largely excluded from lipid rafts.^169^ This exclusion is thought to underline their weaker or qualitatively distinct signaling outcomes. These differences in loading dynamics also dictate which APC subsets drive presentation, fundamentally shaping downstream cytokine cascades. Th1-biasing ligands can be selectively presented by DCs and certain macrophages, triggering a CD40L/CD40-mediated feedback loop in which DC-derived IL-12 recruits NK cells to sustain a second wave of IFN-γ and consolidate the Th1 environment.^167^ Th2-biasing variants, by contrast, are promiscuously loaded onto CD1d by nonprofessional APCs such as B cells, which lack IL-12 secretory capacity, short-circuiting this feedback loop and shifting the response toward Th2 dominance. Additionally, CTLs can upregulate CD1d and present self-lipids to iNKT cells, amplifying IFN-γ production and CTL cytotoxicity independently of classical APCs.^160,170,171^

Aqueous solubility is a further determinant of loading route and activation kinetics. Analogs with short or unsaturated lipid chains (e.g., OCH, C20:2) are sufficiently soluble to load directly onto CD1d at the cell surface, bypassing endosomal trafficking and lipid transfer proteins entirely, which produces early but transient NKT cell activation.^172^ Canonical Th1-biasing ligands are highly hydrophobic by contrast, requiring uptake by serum lipoproteins and subsequent endosomal/lysosomal processing facilitated by lipid transfer proteins such as saposin and GM2 activator protein.^168^

Finally, the glycosidic bond chemistry determines a ligand’s longevity *in vivo*: α-C-glycosides resist α-galactosidase degradation, producing more sustained CD1d presentation and consequently stronger Th1 responses than native O-glycosides.^64^ Stability within the lysosomal compartment is equally important; Th1 ligands remain stably associated with CD1d during endosomal recycling, whereas Th2-biasing variants are frequently displaced by competing endogenous lipids in the acidic lysosomal environment.^173^

Since previous research identified the molecular bases for cytokine polarization,^17–20^ the field has moved beyond basic SAR to embrace more sophisticated therapeutic platforms. Modern medicinal chemistry now utilizes conjugation chemistry and covalent integration to create multifunctional tools, such as peptide-glycolipid vaccines and dual-adjuvant conjugates featuring TLR7/8 or TLR4 agonists. These next-generation self-adjuvanting vaccines ensure cellular colocalization and sustained antigen presentation, addressing earlier challenges in translating cytokine bias from murine models to human clinical applications. The next chapter bridges those foundational SAR insights with the latest advances in targeted delivery systems and masked glycolipid pro-drugs, reflecting a decade of progress in the design of iNKT-cell mediated immunotherapies.

## 4 Therapeutic Potential of CD1d Ligands

In preclinical mouse models, α-GalCer proved to be a highly effective and well-tolerated mucosal adjuvant across intranasal, oral, and sublingual routes, with less concern about iNKT cell anergy than systemic dosing. Yet across more than 30 antitumor trial spanning over two decades of clinical translation, outcomes have been largely disappointing, marked by poor objective responses, profound iNKT cell hypo-responsiveness, and nontrivial toxicities that stand in sharp contrast to preclinical efficacy.^26^ Phase I trial in solid tumors of intravenous injections of α-GalCer were well tolerated up to 4,800 μg/m^2^, but no objective clinical responses were observed, and only a fraction of patients achieved transient stable disease; moreover, circulating iNKT cells typically disappeared from blood within 24 h and patients started with markedly reduced baseline iNKT frequencies, so biological effects depended more on pretreatment iNKT numbers than dose, highlighting both functional exhaustion/anergy and a quantitative deficit in the target population.^70^ Additionally, a randomized placebo-controlled trial for chronic Hepatitis B infection was marred by the fact that 4 out of 27 patients had to discontinue treatment due to rigors. More sophisticated cellular delivery has not fully overcome these issues: in a phase II study of α-GalCer-pulsed APCs as second-line therapy in advanced NSCLC, only 1/35 patients (2.9%) achieved a partial response, 40% had stable disease, and 54.3% progressed, despite clear immunologic activation and a median survival of 21.9 months; importantly, total iNKT cell numbers significantly decreased after treatment, again suggesting treatment-induced quantitative or functional loss of the target compartment.^71^ Even when safety was acceptable, efficacy has been modest: a head-and-neck phase I trial of nasal-submucosal α-GalCer-pulsed APCs reported no serious (≥grade 3) toxicities and some evidence of NK/iNKT activation, but did not document objective tumor regressions in this small cohort of unresectable or recurrent disease.^72^ For these reasons, the first attempts of development of novel analogs mainly aimed to tune potency and cytokine bias, avoiding immune anergy without major mucosal toxicity. Synthetic modifications of α-GalCer enabled the possibility for precise control over immune responses. While dual secretion of both pro- and anti-inflammatory cytokines by α-GalCer-activated iNKT cells often results in antagonistic effects that limit clinical efficacy, rational design of synthetic analogs has been proved to profoundly influence cytokine polarization profiles toward Th2 cytokine production (IL-4, IL-10, IL-13), Th1 (IL-2, IFN-γ), or Th17, with a variety of therapeutic opportunities that span from cancer immunotherapy to autoimmune diseases.

Attempts to enhance potency with more powerful iNKT agonists have exposed a narrow therapeutic window: in healthy volunteers, ABX196 showed strong adjuvant activity for HBV vaccination but produced a limited set of adverse events mechanistically linked to systemic delivery to the liver, with liver NKT activation, IFNγ-driven hepatocyte damage, and consequent hepatotoxicity forcing reformulation strategies to restrict hepatic exposure. In a phase I study combining intramuscular ABX196 with nivolumab in heavily pretreated hepatocellular carcinoma, toxicity was again meaningful: among 10 patients, there were 76 adverse events (95% grade 1–2) including frequent diarrhea, malaise/fatigue, and AST/ALT increases, and although dose-limiting toxicities and treatment-emergent serious adverse events were not seen, objective responses remained rare (1 partial response, overall response rate 10%) with only 4 additional patients achieving stable disease.^150,152^ Derivatives such as N-modified analog DB06-1 (compound **12**, Figure 9) or 7DW8-5 (compound **26**, Figure 13) can be more potent, more Th1-skewed, or better optimized for intranasal delivery than α-GalCer itself.^85^ Nevertheless, in a *C. difficile* toxoid vaccine, Alum/α-GalCer or Alum/7DW8-5 broadened IgG subclasses but did not improve protection over Alum alone, while the Alum/α-GalCer combination uniquely caused transient hepatotoxicity not seen with either component alone, underscoring synergy for toxicity without clear added benefit.^174^ In general, these experiences illustrate a recurring pattern of context-dependent hepatotoxicity driven by a systemic Th1-skewed cytokine burst. For this reason, future CD1d-mediated immunotherapies must focus on maintaining the functionality of iNKT cells over repeated dosing and on delivering the therapy to the appropriate compartments and integrating synergistically with other adjuvants, rather than relying on CD1d agonists as standalone agents.

Yet iNKT cell agonists are potent adjuvants that recruit iNKT cells to serve as universal helpers that bridge innate and adaptive immunity. By recognizing glycolipid antigens presented on the nonpolymorphic CD1d molecule, iNKT cells trigger a rapid activation cascade that enhances both cellular and humoral responses against coadministered antigens across multiple routes of administration, including mucosal delivery.^28^ To overcome their clinical challenges, modern medicinal chemistry techniques are arising for maintaining iNKT cell functionality across multidose administrations through compartmentalized, APC-targeted delivery via nanovectors or chemical conjugation, minimizing off-target systemic exposure. Future strategies should also be able to integrate CD1d agonists synergistically with complementary adjuvants rather than relying on them alone. Such regimens, whether through covalent conjugation or optimized temporal coadministration, can tune the Th1/Th2 balance and enhance the overall clinical viability of iNKT-targeted vaccines.

### 4.1 Combinations of CD1d Ligands with Other Adjuvants

DNA vaccination represents a viable platform for eliciting CTL responses, albeit its efficacy is critically dependent on adjuvant selection. Alum (Alhydrogel) mixed with α-GalCer or 7DW8-5 (compound **26**) as separate components modestly enhanced antigen-specific IgG1/IgG2b and broadened to IgG2c, shifted isotypes toward Th1-associated subclasses, demonstrating immunologic significance of an Alum/CD1d ligand cocktail against *C. difficile*.^174^ 7DW8-5 has been also evaluated in coadministered intramuscular injection with adenoviral PyCSP vaccine leading to colocalization of glycolipid and antigen in draining LNs and DCs, enhancing DC recruitment/activation and stronger CD8^+^ T-cell priming and protection, compared with vaccine alone or α-GalCer.^175^ Simultaneous activation of iNKT cells with α-GalCer-loaded CD1d/anti-HER2 fusion protein and TLR9-triggered DCs with OVA peptide/CpG vaccine produced synergistic DC maturation (MHC-II, CD40, CD86, CD70), about 10-fold higher systemic IL-12 levels, and markedly improved NK and OVA-specific CD8 T-cell responses, leading to stronger tumor control than either alone.^176^ The combined adjuvanticity of the TLR4 agonist monophosphoryl lipid A (MPLA) and α-GalCer was evaluated in the context of an HPV-16 E7 DNA vaccine. Co-administration of α-GalCer and MPLA markedly augmented lymphocyte proliferation, CTL activity, Th1/Th2-associated cytokine production (IFN-γ, IL-4, IL-12), and conferred superior protection against TC-1 tumor challenge compared with either adjuvant alone.^177,178^ Blockade of IL-18 signaling during vaccination significantly attenuated IFN-γ responses and antitumor efficacy, implicating IL-18 as a critical mediator of the synergistic α-GalCer/MPLA adjuvant effect. Another combination that has been explored is the codelivery of TLR4 and TLR7/8 agonists with SARS-CoV-2 RBD on aluminum salts, which is also able to converts Alum’s typical Th2 bias into a Th1- or Th1/Th17-skewed profile while markedly boosting neutralizing antibodies titer.^179^ On a final note, it is worth mentioning that the recent combination of IMM60 (compound **49**) and the PD-1 inhibitor pembrolizumab has successfully harnessed their synergistic potential to activate both innate and adaptive pathways. It is currently being evaluated in phase I/II trials for patients with advanced melanoma and metastatic NSCLC.^141,142^ In these trials, it is formulated within a liposome designated as PORT-2. Early human data indicates that the agonist is well tolerated as monotherapy, with evidence of systemic iNKT and NK cell activation.

### 4.2 Conjugates and Self-Adjuvanting Vaccines

The development of 6″-deoxy-6″-azido-α-GalCer and its reduced counterpart, 6″-deoxy-6″-amino-α-GalCer, have enabled sophisticated chemoselective coupling of α-GalCer to other small molecules or antigens through bio-orthogonal and bioconjugation chemistries.^93^ This chemical versatility has proven particularly valuable for conjugating peptide epitopes to create synthetic vaccines with enhanced B and T cell responses.^174^ For example, click chemistry strategies utilizing 6″-azido-modified α-GalCer have been successfully employed to construct fully synthetic, self-adjuvanting antitumor vaccines through conjugation with tumor-associated MUC1 glycopeptide antigens (compound **64**, Figure 27).^180^ Immunological studies in murine models demonstrate that these vaccines, particularly those featuring diglycosylated MUC1 motifs, effectively trigger the maturation of splenic DCs and macrophages. This activation leads to the production of high levels of antigen-specific IgG antibodies, resulting in a potent complement-dependent cytotoxicity against MUC1-expressing cancer cell lines, such as MCF-7 and B16F10. In the same fashion, αGC-RBD conjugate **65** (Figure 27), prepared by site-specific conjugation of 6″-deoxy-6″-amino-α-GalCer to the N-terminus of SARS-CoV-2 receptor-binding domain protein, demonstrates significantly enhanced immunogenicity compared to unconjugated mixtures.^181^

**Figure 27:**
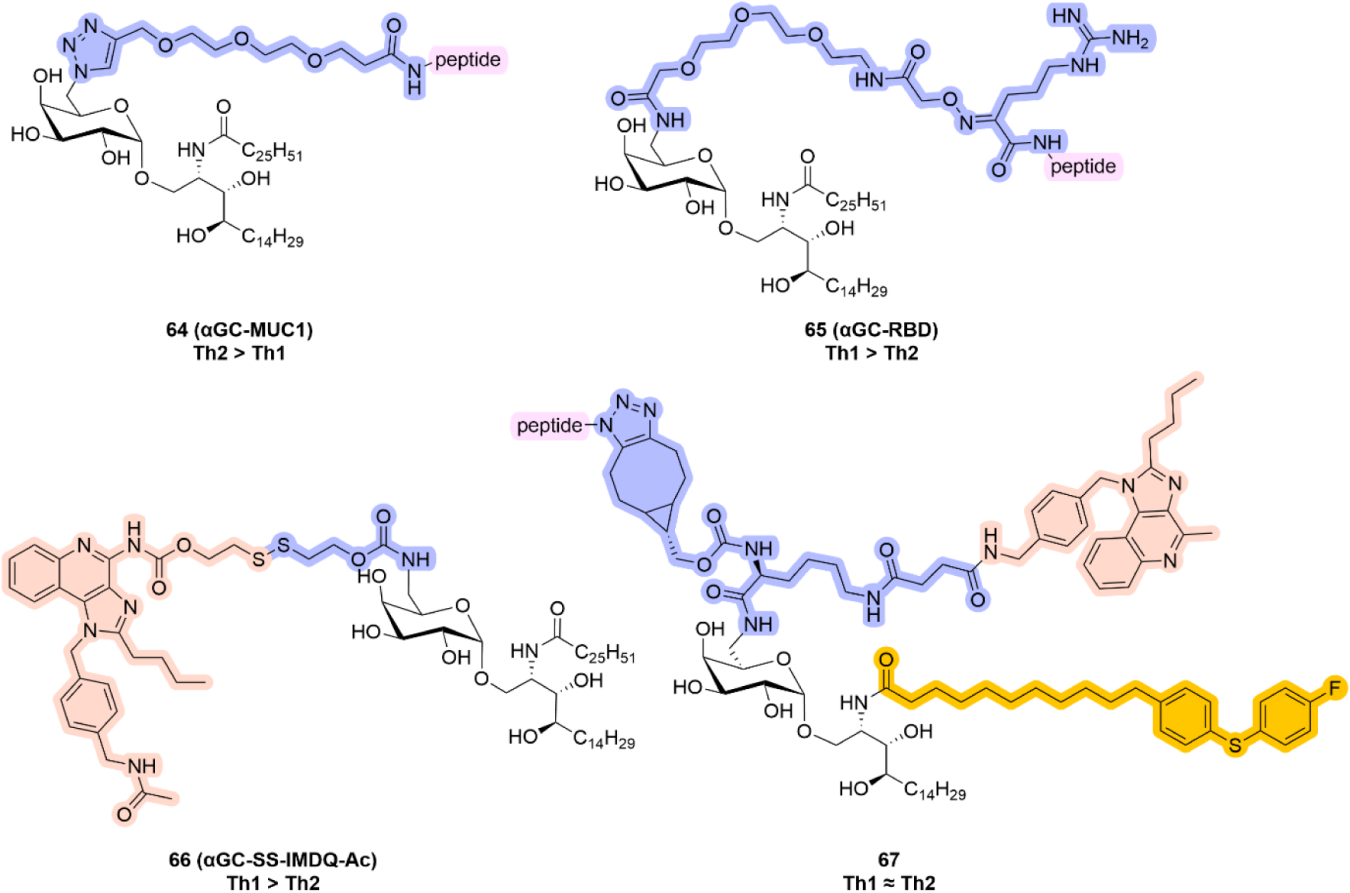
Conjugation approaches in compounds **64**–**67** and their polarization capacity of immune response.

Dual-adjuvant strategies that pair PRR agonists or combine PRR ligands with classical platforms such as αGC-SS-IMDQ-Ac (compound **66**, Figure 27) have repeatedly improved both magnitude and quality of responses, especially against weakly immunogenic protein antigens.^182^ The enhanced activity of covalent conjugates likely results from improved spatial presentation of both CD1d ligands and conventional antigens, facilitating coordinated activation of iNKT cells and conventional T cells within the same immunological synapse. Building on this, the dual conjugate of TLR7/8 and iNKT cell agonists vaccine (compound **67**, Figure 27) was attached through a clickable bicyclononyne to the SARS-CoV-2 RBD protein.^183^ As reported, within this platform, the antigen was able to elicit 20-fold higher levels of IgG2a than control vaccines, highlighting its potential for enhancing antibody-dependent cellular cytotoxicity. This colocalization enhances DC activation and improves antigen cross-presentation, leading to more profound immune responses.

In 2022, a pioneering approach to cancer vaccine design was introduced with the development of a fully synthetic three-component vaccine, MPLA-Tn-α-GalCer (compound **68**, Figure 28), which covalently integrates the tumor-associated carbohydrate antigen (TACA) Tn with two potent immunostimulants, MPLA and α-GalCer.^184^ This innovative construct serves as a self-adjuvanting platform, eliminating the need for external carriers like glycoproteins while harnessing the synergistic activation of innate (via TLR4, although combination with other TLRs are also available)^183^ and adaptive (via iNKT/CD1d) immune pathways. Immunological evaluations in mice revealed that MPLA-Tn-α-GalCer elicited substantial Tn-specific IgG responses, significantly outperforming two-component controls (Tn-MPLA, Tn-α-GalCer) and the traditional glycoprotein conjugate Tn-CRM197, with high-titer antibodies demonstrating specific recognition, binding, and complement-dependent cytotoxicity against Tn-positive cancer cells *in vitro*. *In vivo*, the vaccine markedly enhanced survival rates and extended survival time in tumor-challenged mice, with surviving mice exhibiting durable immunity against subsequent tumor challenges without further treatment. Comparative studies in wild-type and TLR4 knockout mice, alongside CD1d binding affinity assays, confirmed that the covalent linkage of MPLA and α-GalCer drives a synergistic immune activation, amplifying Tn immunogenicity through coordinated TLR4 signaling and iNKT cell-driven cytokine production.

**Figure 28:**
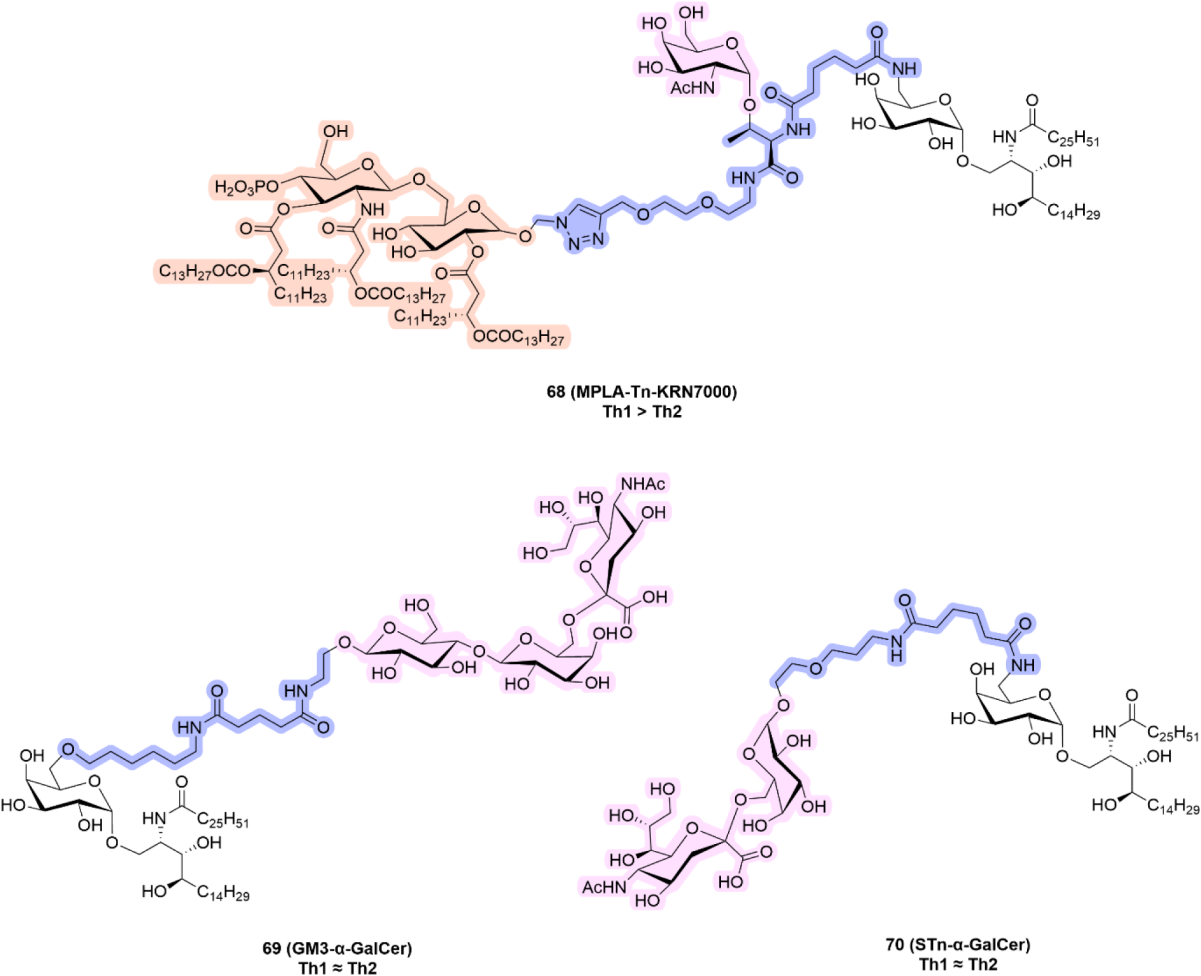
Conjugation approaches in compounds **68**–**70** and their polarization capacity of immune response.

A second fully synthetic tricomponent conjugate (αGC-NP_3_–PADRE) designed to elicit durable antihapten antibody responses was obtained by covalently integrating 6″-amino-6″-deoxy-α-GalCer with a trivalent 4-hydroxy-3-nitrophenyl acetyl (NP) hapten and the universal Pan DR epitope (PADRE) T-helper peptide via an enzymatically cleavable valine-citrulline-para-aminobenzyl (VC-PAB) linker.^185^ In murine models, αGC-NP_3_–PADRE successfully co-opted both innate and adaptive immune pathways and demonstrated several key advantages over its bicomponent (αGC-NP_3_) and the nonconjugated formulations, including significantly greater NKT cell expansion and higher anti-NP IgG antibody levels, robust B cell immunological memory response, and increased affinity over time, which is a hallmark of successful follicular Th-mediated germinal center reactions. The antibody titers generated by the vaccine were found to be comparable to those induced by NP-OVA adjuvanted with Alum.

The conjugation of TACAs to α-GalCer has also been carried out to create fully synthetic self-adjuvanting cancer vaccines. GM3-α-GalCer (compound **69**, Figure 28) and (Neu5Gc)­GM3-α-GalCer constructs demonstrate the ability to induce both Th1 and Th2 cytokines, leading to production of all subclasses of IgG antibodies against tumor-associated glycans.^186^ These conjugates overcome the inherent weak immunogenicity of carbohydrate antigens by providing pronounced T cell support through iNKT cell activation. The balanced Th1/Th2 response ensures that the cytokine response elicits comprehensive immune activation, avoiding excessive bias that could limit therapeutic efficacy against cancer. All the subclasses of IgG antibodies were elicited by this mixed formulation, resulting in the killing of cancer cells via complement-dependent cytotoxicity. GM3-α-GalCer has also been evaluated against a noncovalent liposomal vaccine using an optimized β-GalCer lipid anchor.^187^ The results showed that while both platforms induced high IgG titers, the covalent conjugate (GM3-α-GalCer) was superior at recognizing B16F10 melanoma cells and activating the complement system.

The self-adjuvanting platform STn-α-GalCer (compound **70**, Figure 28) has been reported to elicit substantial IgG antibody responses in mice, demonstrating its potential as a vaccine candidate targeting iNKT cells.^188^ This peptide-free conjugate circumvents the complexities of traditional glycoconjugate vaccines by integrating the glycolipid adjuvant directly into the structure, promoting rapid activation of iNKT cells, cytokine release, and DC maturation. Immunization studies in mice demonstrated exceptional efficacy, with the vaccine inducing a pronounced class switch from STn-specific IgM to high-titer IgG antibodies—up to 20-fold higher than controls—while maintaining specificity for STn-expressing tumor cells, as confirmed by ELISA and glycan microarray analyses. The success of innovative self-adjuvanting vaccines not only addresses the poor immunogenicity of carbohydrate antigens but also paves the way for streamlined, self-adjuvanting platforms in oncology, potentially accelerating translation to clinical candidates for STn-positive cancers like breast and ovarian tumors.

#### 4.2.1 Linker Chemistry Optimization in Self-Adjuvant Vaccines

The efficacy of self-adjuvanting vaccines relies on the bioconjugation techniques utilized to covalently link the peptide antigen to the adjuvant. These linking strategies must satisfy stringent physicochemical criteria: robust stability in systemic circulation, high-yielding biocompatibility during synthesis, and temporally controlled, traceless intracellular release. To this end, researchers have used enzymatically cleavable, self-immolative linkers, most notably cathepsin-sensitive Val-Cit-PAB or Val-Ala motifs, which remain stable in circulation but release the active adjuvant and antigen when they reach endosomal or lysosomal compartments.^189^ A significant advance in the synthesis of these self-adjuvanting conjugates has been the demonstration that the glycolipid/linker is sufficiently robust to withstand the harsh conditions inherent to solid-phase peptide synthesis, including repeated trifluoroacetic acid-mediated deprotection, piperidine treatment, and resin cleavage cocktails. This compatibility has been successfully employed for the synthesis of compound **71** (Figure 29) and allows for an entirely on-resin assembly strategy, wherein the adjuvant is coupled directly to the growing peptide chain, circumventing the need for postsynthetic solution-phase ligation steps that often suffer from poor solubility of the hydrophobic glycolipid, laborious purification, and low conjugation yields.^190^ This not only simplifies the synthetic route and reduces the number of isolated intermediates, but also renders the platform far more amenable to parallel synthesis and iterative medicinal chemistry exploration of antigen/adjuvant combinations, a capability that has historically been a significant bottleneck in the rational development of self-adjuvanting vaccine candidates. In general, shifting the conjugation of peptide antigens from manual wet chemistry to an automated, solid-phase process represents an advancement in the manufacturing of self-adjuvanting immunotherapies, as it enhances efficiency, yield and overall speed of the process.

**Figure 29:**
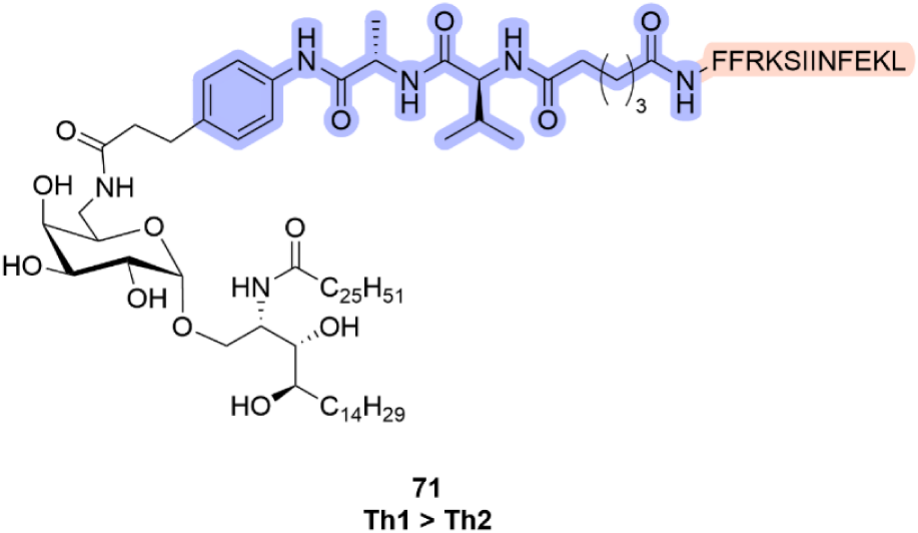
Conjugation approach in compound **71** and its polarization capacity of immune response.

As an alternative to classical click reactions, strain-promoted azide–alkyne cycloaddition (SPAAC) has been employed to eliminate the need for cytotoxic copper catalysts, preventing metal-induced protein denaturation or sample contamination in the preparation of the conjugates.^191^ This technique couples an azide-functionalized antigen with a cycloalkyne-modified adjuvant (e.g., dibenzocyclooctyne). The cycloaddition is driven to rapid completion entirely by the extreme thermodynamic ring strain of the cyclooctyne, yielding a highly stable triazole linkage in purely aqueous media. SPAAC provided exceptionally clean products for long peptides compared to CuAAC, and has been successfully employed for the conjugation of α-GalCer with influenza synthetic long peptides. Oxime condensation has also been employed to generate aminooxy-functionalized component with an aldehyde or ketone to form a highly chemoselective covalent oxime bond, which operates orthogonally to the standard functional groups present in long peptides and is preferred for its small chemical footprint and high efficiency.^192,193^ The resulting oxime linkage possesses greater hydrolytic stability than corresponding hydrazones, providing a robust tether well-suited for multivalent antigen incorporation or integration into liposomal coassemblies. Interestingly, oxime-linked compounds are also more efficiently processed by cathepsin B and induce higher liver-resident memory T cells when employed in self-adjuvanted vaccines involving malaria (NVY, NVF, PbRPL6) and breast cancer (HER2, NY-ESO-1). These conjugates exhibited potent biological activity by specifically enhancing site-specific CD8^+^ T-cell responses. To ensure the precise release of MHC-binding epitopes from the vaccine, incorporation of an N-terminal FFRK sequence is typically employed. This sequence is essential for maximizing liver-resident memory T cell generation and protective immunity, as conjugates lacking it show markedly reduced NKT cell activation and T-cell expansion. Maleimide–thiol strategies (for amine/ester linkers) have also been reported for the synthesis of a self-immolating thiocarbonate to release a thiol-containing agonist, highlighting that secondary amine and ester linkers at the 6″-position showed higher cytokine induction than rigid amide versions.^194,195^

Interestingly, there are reported cases where conjugation is not always the optimal strategy for harnessing iNKT cell adjuvanticity. This is illustrated by studies for smoking cessation on Nic-α-GalCer, a conjugate in which a nicotine hapten is covalently linked to the 6″-position of α-GalCer. The therapeutic rationale underlying both modalities is to generate circulating antibodies that sequester nicotine in the periphery, and the formulation aims to prevent its transit across the blood-brain barrier and thereby blunt its reinforcing effects. In both cases, the resulting immune response displayed a mixed Th1/Th2 profile consistent with the cytokine signature characteristic of the parent α-GalCer scaffold. However, direct comparison with a noncovalent liposomal formulation coincorporating α-GalCer and a lipidated nicotine derivative revealed that the latter was superior in inducing high-titer antinicotine IgG antibodies and in attenuating the pharmacological effects of nicotine, including hypothermia.^196^

### 4.3 Masked Glycolipids and Controlled Activation

Masked α-GalCer (MαGC, **71**, Figure 30) represents an innovative pro-drug approach that enables controlled activation through self-immolative chemistry. The design incorporates a self-immolative linker attached to the N-2 position that undergoes degradation upon uptake by antigen-presenting cells.^189,191,197^ Through O-to-N acyl migration, MαGC is converted to active α-GalCer, regaining full iNKT cell activation capacity. These conjugates stimulated significantly more potent antigen-specific CD8^+^ T cell proliferation and cytotoxic responses in mice compared to simple mixtures of their individual components, with strong Th1-biasing profiles. The therapeutic applications range from the induction of allergen- or hapten-specific CTLs to promote a T cell memory pool dominated by effector memory T cells phenotype, to peptide-specific CTLs against cells expressing viral oncoprotein, as in α-GalCer-pp65 and α-GalCer-E7. This controlled activation strategy offers potential advantages for tissue-specific targeting and reduced systemic toxicity, as the pro-drug remains inactive until processed by appropriate cells. The self-immolative mechanism ensures efficient conversion to the active form while maintaining the structural integrity required for CD1d binding and presentation. Pro-drug approaches may be particularly valuable for cancer immunotherapy applications where localized immune activation is desired while minimizing systemic immune stimulation. The success of MαGC validates chemical approaches to controlled drug release and provides a framework for developing additional pro-drug strategies.

**Figure 30:**
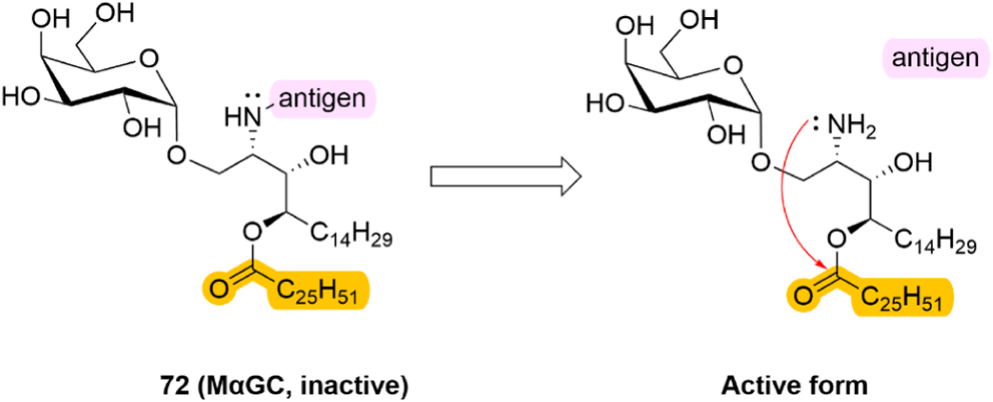
Structure of masked α-GalCer **72** precursor and its mechanism of activation.

### 4.4 Application of α-GalCer Derivatives as Probes

Some effort in the derivatization of α-GalCer has also been directed into designing investigative tools, allowing us to study the crystallographic properties of the binding to CD1d, visualize the iNKT cell activation, and control the activation moment. For example, bromine-containing derivative at the acyl termini **73** (Figure 31) has been studied for its application as crystallographic probe as Br and a methyl group possess similar van der Waals radii (Br: 185 pm; CH_3_: 200 pm) and preserves essential hydrophobic interactions in the ligand-protein complex.^198^ Similarly, the selenoether group in compound **74** (Figure 31) maintains comparable bond angles (C–Se–C: 96.3°; C–C–C: 112.6°) and van der Waals radii (Se: 190 pm; CH_2_: 200 pm) to carbon, despite a slightly larger bond length (C–Se: 194.5 pm; C–C: 154.0 pm). Notably, these structural modifications have been reported to skew cytokine responses toward a Th1-biased profile, enhancing IFN-γ secretion in murine spleen cells.

**Figure 31:**
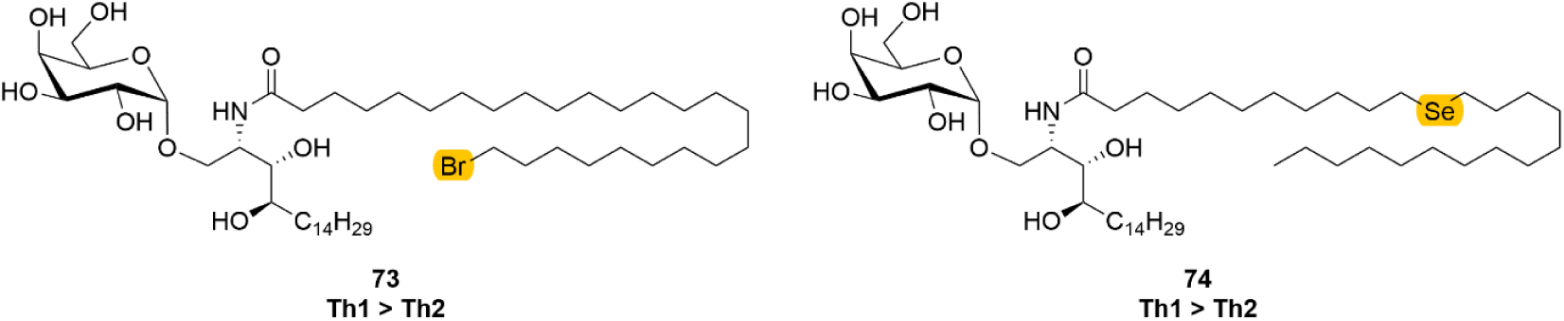
Structural modifications in compounds **73** and **74** and their polarization capacity of immune response.

The development of fluorescent CD1d ligands has provided essential tools in an attempt to track the *in vivo* uptake, trafficking and presentation mechanism of CD1d-restricted antigens. Early efforts focused on C6″-dansylated α-GalCer, which maintains iNKT cell activation capacity similar to the parent compound while enabling fluorescence-based detection (compound **75**, Figure 32).^199^ However, dansyl groups suffer from limitations including modest quantum yields and environmental sensitivity. BODIPY-α-GalCer (compound **76**, Figure 32) represents a significant advancement in fluorescent probe design, offering superior photophysical properties including high fluorescent quantum yield, large extinction coefficient, and photostability compared to dansyl derivatives.^200^ The compact size of the BODIPY fluorophore minimizes steric perturbations while providing excellent detection sensitivity, making it an ideal probe for mechanistic studies and high-throughput screening applications. These fluorescent probes have enabled detailed studies of CD1d trafficking, ligand presentation kinetics, and cellular localization, contributing to fundamental understanding of the CD1d antigen presentation pathway. The availability of high-quality fluorescent ligands continues to drive advances in both basic research and diagnostic applications.

**Figure 32:**
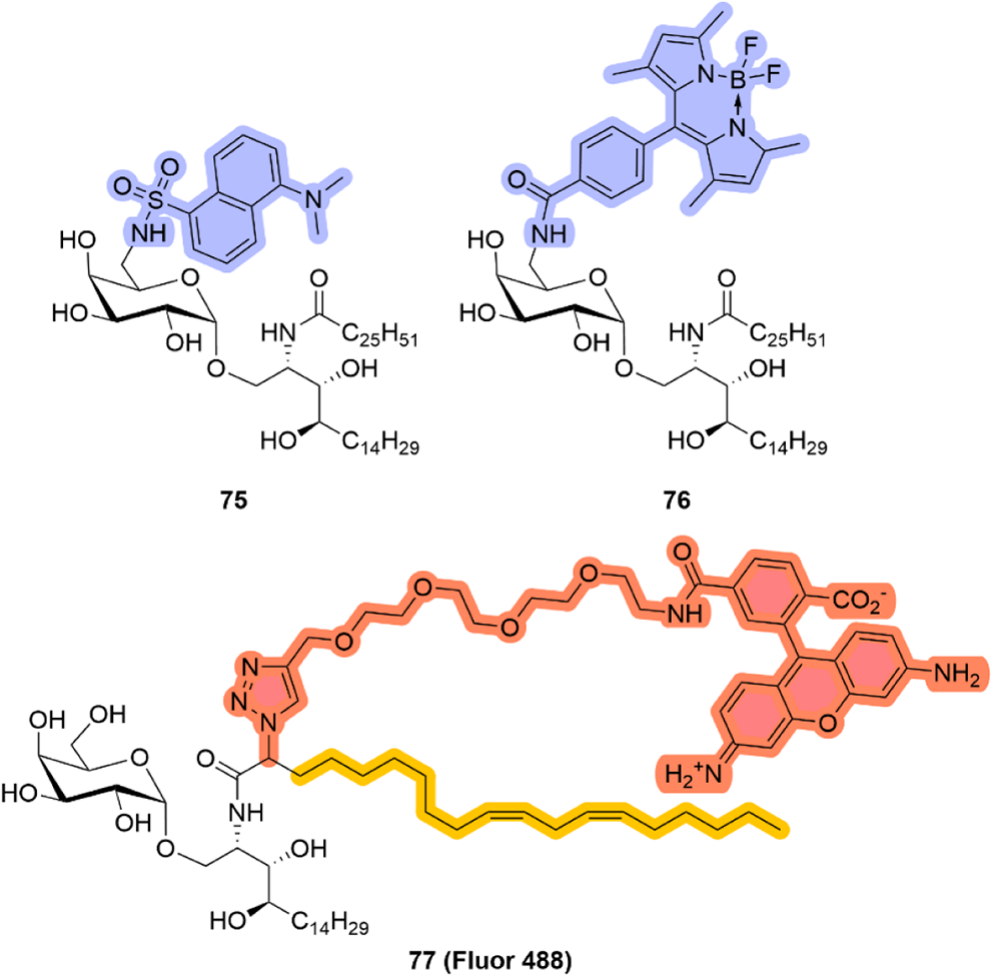
Structural modifications of labeled glycolipid analogs **75, 76** and **77**.

Recently, biotin and fluorescent reporter labels (such as in derivative Fluor 488, Figure 32, compound **77**) have been appended to CD1d ligands. X-ray crystallographic analysis of the TCR/glycolipid/CD1d ternary complex revealed that substitution of the α-methylene group in the acyl chain causes it to protrude away from the binding site toward the solvent interface, ensuring the modifications have minimal impact on the agonist’s functionality.^172^ These probes remain functionally active and accessible for recognition by streptavidin or antibodies, even when bound within the ternary complex, thus providing a viable platform for studying antigen uptake and loading mechanisms.

Finally, photoswitchable glycolipid analogs such as compound **78** (Figure 33) provide a valuable tool to investigate spatiotemporal control of cytokine production through optical activation. The introduction of an azobenzene moiety into the acyl chain of α-GalCer elicits very low cytokine production in its *trans* configuration but triggers significant IL-4 and IFN-γ secretion following irradiation at 370 and 440 nm (reversible *trans*/*cis* isomerization).^201^ Photochromic galactosylceramides could be used as probes for understanding the cellular and molecular basis of CD1d-restricted antigen presentation and provide a conceptual basis for photoimmunotherapeutic applications.

**Figure 33:**
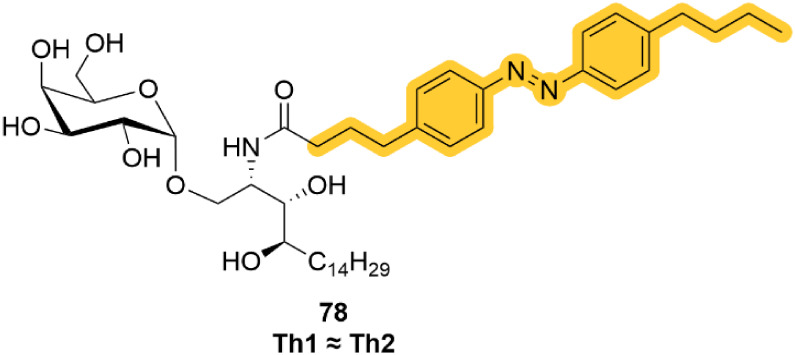
Structure of the photoswitchable glycolipid analog **78** and its polarization capacity of immune response.

## 5 Conclusions and Future Perspectives

The evolution of CD1d-restricted glycolipids from the prototypical α-GalCer to sophisticated, tailored immunotherapeutics represents a significant leap in medicinal chemistry. Over the last two decades, extensive SAR studies have transitioned the field from simple ligand discovery toward the engineering of multifunctional therapeutic platforms capable of orchestrating precise innate and adaptive immune responses. While α-GalCer remains a potent benchmark, its clinical utility has been hampered by its poor solubility and the induction of long-term iNKT cell anergy. Modern structural innovations, including branched *N*-alkyl derivatives and PEGylated functionalities, have directly addressed these challenges to enhance aqueous solubility, facilitating easier formulation and alternative administration routes like mucosal delivery. In addition, novel scaffolds have been developed to avoid the long-term functional exhaustion associated with α-GalCer, allowing iNKT cells to recover their cytokine-producing capacity. Finally, the development of masked or photoswitchable glycolipids provides a pro-drug strategy for controlled, tissue-specific activation, potentially reducing systemic toxicity and the risk of cytokine storms.

The ability to rationally design ligands with defined Th1- or Th2-skewing profiles constitutes a promising expansion of the medicinal chemist’s toolkit and introduces tangible clinical opportunities. Th1-biased responses, desirable in oncology and infectious disease settings, have been achieved through strategic aromatic modification at the acyl terminus, substitution at the C6″ position of the galactose headgroup, and incorporation of thioamide bioisosteres that enhance stabilization of the CD1d/ligand/TCR ternary complex. These interventions prolong antigen presentation and reinforce IFN-γ-dominant cytokine output. Conversely, truncation or unsaturation of the lipid chains reduces overall complex stability, thereby promoting more transient CD1d engagement and preferential early IL-4 secretion.

The most promising frontier lies in fully synthetic, self-adjuvanting vaccines. Covalent conjugation of glycolipid agonists to defined antigens, such as the SARS-CoV-2 RBD peptide or TACAs enables coordinated delivery and colocalization to the same immunological synapses, ensuring that ensures that the CD1d-mediated iNKT activation and antigen presentation occur in a spatially and temporally synchronized manner. These conjugates overcome the weak immunogenicity of carbohydrates, inducing high-titer, high-affinity IgG responses, while amplifying immune activation through convergent innate and adaptive pathways.

The next phase of CD1d-mediated immunotherapy is likely to prioritize rationally designed synergistic regimens. The clinical evaluation of CD1d ligands in combination with pembrolizumab exemplifies this concept, highlighting the therapeutic potential of integrating innate immune activation with inhibitory pathway release. Ultimately, the evolution from broad-spectrum immune stimulation toward structurally defined, stable, and pharmacologically tractable glycolipid architectures positions CD1d ligands as a cornerstone of precision immunotherapy across oncology and vaccine development.

## Abbreviations

α-GalCer: α-galactosylceramide (αGC, KRN7000)
α-GlcCer: α-glucosylceramide
AdPyCS: adenoviral Plasmodium yoelii circumsporozoite protein vaccine
Alum: aluminum salts adjuvant
AP-1: activator protein 1
APC: antigen-presenting cell
AraCer: arabinitolceramide
BCN: bicyclononyne
BODIPY: boron-dipyrromethene fluorophore
CD: cluster of differentiation
CD40L: CD40 ligand
CDC: complement-dependent cytotoxicity
CDR: complementarity-determining region
CRM197: cross-reacting material 197
CTL: cytotoxic T lymphocyte
CuAAC: copper-catalyzed azide–alkyne cycloaddition
DC: dendritic cell
DMSO: dimethyl sulfoxide
EAE: experimental autoimmune encephalomyelitis
ELISA: enzyme-linked immunosorbent assay
Fas: Fas cell surface death receptor
FasL: Fas ligand
Fc: fragment crystallizable
FOXP3: forkhead box P3
GM3: monosialodihexosyl ganglioside
GlyCer: glycerolceramide
GrB: granzyme B
HBs: Hepatitis B surface antigen
HPV: human papillomavirus
IBD: inflammatory bowel disease
IFN: interferon
iGb3: Isoglobotrihexosylceramide
IgG: immunoglobulin G
IgM: immunoglobulin M
IL: interleukin
IMDQ: imidazoquinoline
Inkt: invariant natural killer T
IRF: interferon regulatory factor
ITAM: immunoreceptor tyrosine-based activation motif
LAT: linker for activation of T cells
Lck: lymphocyte-specific protein tyrosine kinase
LNP: lipid-nanoparticle
MαGC: masked α-galactosylceramide
MALDI-TOF: matrix-assisted laser desorption/ionization-time-of-flight
MAPK: mitogen-activated protein kinase
MD: molecular dynamics
MDSC: myeloid-derived suppressor cell
MHC: major histocompatibility complex
Mincle: macrophage-inducible C-type lectin
MPLA: monophosphoryl lipid A
MyD88: myeloid differentiation primary response 88
Neu5Gc: N-glycolylneuraminic acid
NFAT: nuclear factor of activated T cells
NF-κB: nuclear factor kappa-light-chain-enhancer of activated B cells
NK: natural killer
NOS: nitric oxide synthase
OVA: ovalbumin
NSCLC: nonsmall cell lung cancer
PBMCs: peripheral blood mononuclear cells
PD-1: programmed cell death protein 1
PDB: Protein Data Bank
PKC: protein kinase C
PLCγ: phospholipase C gamma
PRR: pattern recognition receptor
PyCSP: Plasmodium yoelii circumsporozoite protein
RBD: receptor-binding domain
SAR: structure–activity relationship
SARS-CoV-2: severe acute respiratory syndrome coronavirus 2
SDS-PAGE: sodium dodecyl sulfate-polyacrylamide gel electrophoresis
SPAAC: strain-promoted azide–alkyne cycloaddition
STn: sialyl-Tn antigen
TACA: tumor-associated carbohydrate antigen
TCR: T-cell receptor
TC-1: HPV-16 E6/E7-expressing murine tumor cell line;
Th: T helper
TLR: Toll-like receptor
TME: tumor microenvironment
TNF-α: tumor necrosis factor-α
Tn: GalNAc-α-O-Ser/Thr antigen
TRAF: TNF receptor-associated factor
Treg: regulatory T cell
ThrCer: threitolceramide
VC-PAB: valine-citrulline-para-aminobenzyl
ZAP-70: zeta-chain-associated protein kinase 70
